# Allostatic load, genetic susceptibility, and lung cancer risk: Evidence from a prospective cohort and integrative proteomic analyses

**DOI:** 10.1371/journal.pdig.0001766

**Published:** 2026-10-14

**Authors:** Fan Yang, Wei Wang, Fangyi Chen, Zhichao Wang, Yifan Wang, Tianchen Qi, Zhazheng He, Shuang Liang, Qingyin Bu, Xirui Qiu, Dong Jiaqing, Shuai Ren, Juncheng Dai, Xiaoxiao Wang

**Affiliations:** 1 Department of Epidemiology, Center for Global Health, School of Public Health, Nanjing Medical University, Nanjing, China; 2 GCP Center, Affiliated Hospital of Nanjing University of Chinese Medicine, Nanjing, China; 3 Clinical Research Institut, Affiliated Hospital of Nanjing University of Chinese Medicine, Nanjing, China; 4 Department of Respiratory and Critical Medicine, Affiliated Hospital of Nanjing University of Chinese Medicine (Jiangsu Hospital of Traditional Chinese Medicine), Nanjing, China; 5 Department of Radiology, Affiliated Hospital of Nanjing University of Chinese Medicine, Nanjing, China; 6 Jiangsu Key Lab of Cancer Biomarkers, Prevention and Treatment, Collaborative Innovation Center for Cancer Personalized Medicine and China International Cooperation Center for Environment and Human Health, Gusu School, Nanjing Medical University, Nanjing, Jiangsu, China; University of Chicago Pritzker School of Medicine, UNITED STATES OF AMERICA

## Abstract

Allostatic load (AL), a composite measure of multisystem dysregulation associated with chronic stress, has been linked to lung cancer risk. However, its joint effects with genetic susceptibility and the biological pathways underlying this relationship remain unclear. We conducted a prospective cohort study of 336,945 UK Biobank participants to assess associations between AL and lung cancer incidence and its histological subtypes. A polygenic risk score was constructed to evaluate the joint associations of AL and genetic susceptibility. We further assessed whether AL provided incremental risk stratification beyond established low-dose computed tomography (LDCT) screening eligibility frameworks and integrated plasma proteomic data using machine learning, enrichment, and mediation analyses. We found that higher AL was associated with an increased risk of lung cancer (per-unit HR = 1.073, 95% CI: 1.053–1.094; Low vs. Normal HR = 1.284, 95% CI: 1.065–1.547; High vs. Normal HR = 1.629, 95% CI: 1.347–1.971). Subtype analyses revealed the strongest effect for small-cell lung cancer (SCLC), with significant associations also observed for lung adenocarcinoma and squamous cell carcinoma. High AL combined with high genetic risk was associated with a 136.9% increased risk (HR = 2.369, 95% CI: 1.565–3.583), without evidence of interaction. AL provided additional risk discrimination beyond established LDCT screening eligibility criteria. Proteomic analyses identified 111 AL-associated proteins and 34 proteins with potential mediation signal, including HGF, RARRES2, and GDF15, implicating inflammatory, metabolic, and growth factor-related processes as biological correlates of the AL–lung cancer association. In conclusion, higher AL was independently associated with lung cancer risk, particularly SCLC. Integrative proteomic analyses provided exploratory evidence for biological pathways that may link cumulative physiological dysregulation to lung carcinogenesis. Incorporating AL, as a composite biomarker reflecting cumulative physiological burden, into risk models may improve risk stratification and help identify individuals at elevated risk.

## Author summary

Lung cancer remains the leading cause of cancer-related death worldwide. We investigated whether long-term physiological stress, quantified using a measure known as allostatic load that captures cumulative dysregulation across multiple body systems, is associated with the development of lung cancer. We also examined whether AL and inherited genetic susceptibility were jointly associated with lung cancer risk. Using data from a large prospective population cohort, we found that individuals with higher levels of physiological stress had a substantially greater risk of developing lung cancer. The association was particularly strong for small-cell lung cancer, an aggressive subtype. People with both high stress burden and high genetic risk experienced the highest incidence rates, although the two factors appeared to contribute independently. We also found that allostatic load provided additional risk stratification beyond existing low-dose CT screening eligibility criteria, suggesting that it may help identify high-risk individuals not fully captured by current screening rules. To explore potential biological explanations, we integrated large-scale plasma protein data and identified stress-related inflammatory and metabolic pathways that may connect chronic physiological dysregulation to lung carcinogenesis. Our findings suggest that long-term systemic stress, as captured by allostatic load, is associated with lung cancer development and may reflect cumulative biological and behavioral disadvantage. Incorporating stress-related biological measures into risk assessment may improve identification of high-risk individuals and inform prevention strategies.

## 1. Introduction

Lung cancer remains the leading cause of cancer-related mortality worldwide, accounting for 12.4% of new cancer cases and 18.7% of cancer deaths annually [1]. Despite advances in early detection and treatment, its prognosis remains poor, primarily due to late-stage diagnosis and the complex, multifactorial nature of its etiology [2]. Although smoking is the most well-established risk factor for lung cancer, about 10%–20% of lung cancer patients are never-smokers [3,4], highlighting the critical need to identify additional determinants to inform effective prevention strategies. Moreover, lung cancer subtypes differ substantially in pathogenesis, risk profiles, therapeutic responses, and prognoses. Small-cell lung cancer (SCLC) is characterized by rapid growth, early metastasis, and poor outcomes [5], whereas non-small-cell lung cancer (NSCLC) encompasses multiple subtypes that arise through more diverse etiological pathways and generally exhibit a more favorable prognosis [6]. Therefore, systematic investigation of additional risk markers and factors beyond smoking, particularly their associations with specific lung cancer subtypes, is warranted.

Chronic stress is increasingly recognized as a contributor to multisystem physiological dysregulation and has been implicated in adverse health outcomes, including cancer [7]. Allostatic load (AL), a widely used and validated composite index quantifying the cumulative biological burden of chronic stress, captures long-term wear and tear across neuroendocrine, immune, metabolic, and cardiovascular systems [8]. As an integrative measure of physiological dysregulation, AL may also reflect long-term exposure to behavioral, socioeconomic, and environmental disadvantages. Elevated AL has been associated with frailty, neurodegenerative disorders, cardiovascular disease, and all-cause mortality [9]. Emerging evidence links high AL to increased risk of breast and colorectal cancer as well as cancer-related mortality [10–12]. A recent study further indicates that elevated AL is associated with a higher risk of lung cancer, particularly among former and current smokers [13]. However, whether AL jointly interacts with other risk factors and whether the impact of AL varies by subtype remains unclear.

Genetic alterations represent a pivotal role in the development of lung cancer [14]. Large-scale genome-wide association studies (GWAS) have identified multiple susceptibility loci associated with lung cancer risk [15]. Although individual variants contribute modest risk, aggregating them into a polygenic risk score (PRS) enhances the ability to identify individuals at elevated risk [16,17]. In a European ancestry population, a PRS based on 128 single-nucleotide polymorphisms (SNPs) effectively distinguished high- versus low-risk individuals [18]. Moreover, previous studies indicate that PRS interacts with environmental and lifestyle factors, resulting in variable genetic risk effects across different exposure levels [19]. Yet, the potential interaction between AL and PRS in modulating lung cancer risk remains unexplored, limiting our understanding of how inherited genetic risk and sustained physiological stress jointly relate to lung cancer risk.

Despite the growing evidence linking AL to lung cancer risk, the biological correlates of this association remain poorly understood. The development of lung cancer is known to correlate with alterations in circulating proteins [11]. Plasma proteins, which reflect systemic physiological and pathological states, represent a promising resource for biomarker discovery and may offer clues to disease-related biology [20]. Given that AL represents cumulative dysregulation across metabolic, inflammatory, immune, and endocrine systems, plasma proteomic profiles may provide a useful intermediate phenotype for characterizing biological alterations associated with higher AL. However, evidence connecting plasma protein alterations with elevated AL remains limited in the context of lung cancer.

To address these knowledge gaps, we conducted a prospective cohort study of 336,945 participants derived from UK Biobank (UKB) to investigate the associations of AL with lung cancer incidence, both overall and by major histological subtypes, and to examine the joint contribution of AL and genetic susceptibility to lung cancer risk. The potential clinical relevance of AL for lung cancer screening was evaluated by assessing whether AL could refine risk stratification within established low-dose computed tomography (LDCT) screening eligibility frameworks, including the United States Preventive Services Task Force (USPSTF) 2021 and National Lung Screening Trial (NLST) criteria, and whether incorporating AL improved model discrimination beyond rule-based screening criteria. We further integrated plasma proteomics to identify proteins associated with elevated AL and to explore their potential roles in pathways that may connect AL with lung cancer risk. Through this integrative strategy, we aim to extend previous evidence on AL and lung cancer from epidemiological association toward screening-oriented risk stratification and biological interpretation at the intersection of chronic stress biology, genetic predisposition, and circulating proteomic alterations.

## 2. Materials and methods

### 2.1 Study design and participants

The UKB is a large-scale prospective population-based cohort study launched in 2006, which recruited approximately 500,000 individuals aged 40–69 years across 22 assessment centers in England, Wales, and Scotland, with comprehensive data collection on demographics, lifestyle, and biological samples for diverse health-related analyses [21]. The UKB database received approval from the Northwest Multi-Center Research Ethics Committee. All participants in the UKB provided written informed consent. The UK Biobank study was approved by the North West Multicenter Research Ethics Committee, and all participants provided written informed consent. The present analysis was conducted under UK Biobank application number 79151.

The study design and flowchart are shown in S1 Fig. We excluded 126,829 individuals with missing biomarkers constituting the AL index, as well as 17,255 individuals with cancer at baseline. 21,367 individuals lacking PRS were also excluded. Finally, 336,945 individuals in the UK Biobank were included (S1A Fig). In parallel, we also incorporated data from the UK Biobank Pharma Proteomics Project (UKB PPP), which includes 53,073 participants with available plasma proteomic and biomarker measurements. After excluding participants with a cancer diagnosis at baseline (n = 4,417) and those without complete AL biomarker data (n = 12,497), 36,159 participants with both complete AL biomarker profiles and plasma protein measurements were retained for the mediation analysis (S1B Fig).

### 2.2 Plasma proteomics

During 2006–2010, baseline blood samples were collected from participants at recruitment. Between 2021 and 2022, the UKB-PPP conducted proteomic profiling on these plasma samples from approximately 53,073 UKB participants using the Olink™ Explore 3072 proximity extension assay, measuring 2,941 protein analytes and identifying 2,923 unique proteins. Proteins were measured across eight disease-focused panels, including Cardiometabolic I & II, Inflammation I & II, Neurology I & II, and Oncology I & II. A rigorous quality-control pipeline was implemented, including sample selection, outlier and swap detection, and removal of flagged assays; details are available in prior methodological reports [22]. Protein abundances were normalized to log_2_-scaled Normalized Protein eXpression (NPX) values [23]. GLTPR1 was excluded because over 80% of its data failed quality control [22]. 11 proteins missing over 20% were excluded, resulting in a total of 2,911 proteins included in further analysis. K-nearest-neighbor algorithm was applied to impute proteins with missing values less than 20% and inverse NPX transformation was applied on imputed proteins [17].

### 2.3 Construction of allostatic load score

The development of AL score was guided by established methods reported in previous studies [12]. Briefly, AL score was comprised of 12 biomarkers across four physiological systems, all of which are available in UKB dataset: 1) metabolic system: glycated hemoglobin (HbA1c), high-density lipoprotein cholesterol (HDL), low-density lipoprotein cholesterol (LDL), total cholesterol (TC), triglycerides (TG), waist-to-hip ratio (WHR); 2) cardiovascular system: systolic and diastolic blood pressure (SBP, and DBP, respectively), pulse rate (PR); 3) inflammatory or immune system: C-reactive protein (CRP), and insulin-like growth factor-1 (IGF-1); 4) kidney function: creatinine (Cre). For each of 12 biomarkers, all participants were classified into quartiles based on the distribution of the values of biomarker at baseline. Specifically, the lowest quartile was defined as the high-risk range for HDL cholesterol and IGF-1, while the highest quartile was designated as high-risk for the remaining ten biomarkers. A score of 1 was assigned if a biomarker value fell within the high-risk quartile, and 0 otherwise. The total score thus ranged from 0 to 12, with higher score indicating greater physiological dysregulation associated with chronic stress.

To evaluate the association between AL and clinical outcomes, two strategies were employed. First, the AL score was analyzed as a continuous variable to assess dose-response relationship. Second, AL was analyzed as a categorical variable. Prior literature dichotomized the AL score into low and high-risk groups using the median as the cut-off [12]. Considering that a score of 0 indicates no biomarker abnormality, we further refined this by classifying AL burden into three categories: normal (0), low (1 to median) and high (> median), corresponding to no, modest and substantial biomarker abnormalities, respectively.

### 2.4 Approaches for deriving polygenic risk score

The PRS for lung cancer was derived using 128 SNPs identified on International Lung Cancer Consortium (ILCCO) data and subsequently applied to UK Biobank [20] (S1 Table). An additive model was applied, where each participant’s PRS was calculated as the weighted sum of risk alleles, with weights corresponding to the coefficients estimated by penalized regression with 10-fold cross-validation on the ILCCO training data [24]. Based on PRS distribution among individuals without lung cancer, participants were classified into three genetic risk categories: low (first quartile), intermediate (second and third quartiles), and high (fourth quartile), as described previously [24].

### 2.5 Outcomes assessments

The primary outcome of this study was lung cancer incidence. The complete dates of follow-up for cancer registry data were 31 December 2020 for England, 31 December 2016 for Wales, and 30 November 2021 for Scotland. All diseases were confirmed according to the 10th Revision of the International Classification of Diseases (ICD-10), and ICD-10 codes of lung cancer were defined as C33–C34. Based on histopathological classification, lung cancer was first categorized into SCLC and NSCLC. Within NSCLC, we further identified three major histological subtypes: lung adenocarcinoma (LUAD), squamous cell lung carcinoma (LUSC), and large cell carcinoma (LCC), according to the International Classification of Diseases for Oncology, Third Edition (ICD-O-3). The date and cause of death were obtained from death certificates held within the National Health Service Information Centre (England and Wales) and the National Health Service Central Register (Scotland). Baseline was defined as the date of UK Biobank enrollment, when exposures and covariates were assessed. Participants were followed from baseline until the diagnosis of lung cancer or censoring, defined as the earliest occurrence of death, withdrawal from the study, or administrative end of follow-up.

### 2.6 Covariates

Potential confounders were selected based on prior literature and biological plausibility. The covariates included in this study were baseline age, sex (male, and female), ethnicity (non-European population, and European population), body mass index (BMI), smoking status (never, former, and current), frequency of alcohol intake (daily or almost daily, three or four times a week, once or twice a week, one to three times a month, special occasions only, and never), International Physical Activity Questionnaire (IPAQ) activity group (high, moderate, and low), education history (have a college or university degree and other), Townsend deprivation index (TDI), and family history of lung cancer (yes, and no).

To address missing data, a missing indicator approach was applied for categorical variables, while continuous variables were imputed using sex-specific medians. Overall, missingness was minimal (< 2%) across covariates, apart from IPAQ, which exhibited a missing rate of 18.83%.

### 2.7 Statistical analysis

For descriptive analysis, continuous variables were presented as mean with standard deviation (SD) or median with interquartile range (IQR), and categorical variables were summarized as counts with corresponding percentages. Differences between lung cancer free individuals and those with lung cancer were assessed using Student’s t-test or Wilcoxon rank sum test for continuous variables, and chi-square test or Fisher’s exact test for categorical variables as appropriate.

Cox proportional hazard models were applied to investigate the impact of AL on both lung cancer and lung cancer subtypes, adjusting for age, sex, BMI, smoking status, alcohol intake frequency, IPAQ, education level, TDI and family history of lung cancer. In addition, to disentangle the contribution of individual physiological domains, we further examined the associations between lung cancer risk and AL subsystems using the same modeling strategy. The proportional hazards assumption was assessed using Schoenfeld residuals, with no evidence of violation observed. Results were presented as hazard ratio (HR) with 95% confidence interval (CI). Kaplan–Meier curves with log-rank test were conducted to compare lung cancer incidence among different levels of AL. Potential non-linear associations between AL score and lung cancer risk were evaluated using restricted cubic spline models within the Cox regression framework.

Additive interactions between AL and genetic susceptibility were assessed using an interaction model across combinations of AL and PRS levels, with individuals having both low AL and low PRS defined as the reference group. The additive interaction was evaluated by using three indexes: the relative excess risk due to the interaction (RERI), the attributable proportion due to the interaction (AP) and Synergy Index (SI). The formula was defined as follows:


$$\begin{array}{c} RERI\;=\;HR_{\text{1}\text{1}}-\;HR_{\text{1}\text{0}}-\;HR_{\text{0}\text{1}}+\;\text{1} \end{array}$$

$$\begin{array}{c} AP\;=\frac{RERI}{HR_{\text{1}\text{1}}} \end{array}$$

$$\begin{array}{c} SI\;=\frac{HR_{\text{1}\text{1}}-\;\text{1}}{\left(HR_{\text{1}\text{0}}-\;\text{1}\right)+\;\left(HR_{\text{0}\text{1}}-\;\text{1}\right)} \end{array}$$

HR_11_ represents the hazard ratio for the joint exposure category of interest, HR_10_ and HR_01_ represent the hazard ratios for each corresponding single-exposure category, and HR_00_ represents the reference group with both low AL and low PRS and was set to 1. Corresponding 95% CIs were calculated. The absence of additive interaction was defined as a 95% CI including 0 for RERI and AP, and including 1 for SI. Multiplicative interaction was additionally assessed by including a cross-product term in the Cox model, and the corresponding interaction HR was reported.

To evaluate the relevance of AL for LDCT screening risk stratification, we constructed screening eligibility variables according to the USPSTF 2021 and NLST criteria. USPSTF 2021 eligibility was defined as age 50–80 years, a smoking history of at least 20 pack-years, and current smoking or smoking cessation within the past 15 years [25]. NLST eligibility was defined as age 55–74 years, a smoking history of at least 30 pack-years, and current smoking or smoking cessation within the past 15 years [26]. Participants who did not meet the corresponding eligibility criteria were classified as screening-ineligible. To determine whether AL could refine risk stratification beyond rule-based LDCT screening eligibility, we evaluated the association between continuous AL score and lung cancer risk separately within screening-eligible and screening-ineligible groups. We further cross-classified participants according to screening eligibility status and AL category, using screening-ineligible individuals with normal AL as the reference group. Cox proportional hazards models were then used to estimate hazard ratios and 95% confidence intervals across joint exposure categories, with adjustment for the same covariates as in the primary analysis. To assess whether AL improved risk discrimination beyond established LDCT screening eligibility criteria, we compared Cox models including screening eligibility alone with models including screening eligibility plus AL. Model discrimination was evaluated using Harrell’s C-index, and changes in C-index were calculated by subtracting the C-index of the base model from that of the AL-enhanced model. In parallel, we evaluated the incremental value of AL beyond a multivariable clinical model that included demographic, clinical, and smoking-related covariates. Model improvement was assessed using likelihood ratio tests, and two-sided P values were reported.

In addition to the above primary analysis, we also conducted several supplementary analyses to enhance the comprehensiveness of our study. Subgroup analyses of AL and lung cancer risk were conducted by stratifying participants by the covariates. Given the strong etiological role of smoking in lung cancer, we further examined whether smoking status modified the association between AL and lung cancer risk by including a multiplicative interaction term between AL and smoking status in the Cox proportional hazards models. Furthermore, several sensitivity analyses were conducted to examine the robustness of the results. First, we excluded participants with lung cancer events within the first year from baseline. Second, we excluded participants with death events in the first 3 years of follow-up. Third, we additionally adjusted for detailed smoking-related variables, including smoking duration, number of cigarettes smoked per day, and years since quitting smoking, in analyses of both overall lung cancer and histological subtypes. Fourth, we excluded participants with chronic obstructive pulmonary disease (COPD) at baseline. Fifth, given the potential bias in Cox regression with few events, Firth’s penalized Cox regression was applied to assess the association between AL and lung cancer subtypes, using the same adjustment covariates as the primary analysis.

Plasma proteins associated with AL were identified using a machine learning framework. An eXtreme Gradient Boosting (XGBoost) regression model was trained to predict AL based on 2,911 plasma proteins, with hyperparameters optimized via the Optuna framework using five-fold cross-validation to minimize mean squared error. Feature selection was performed using the Boruta-SHAP algorithm, which integrates permutation-based shadow features with SHAP values to identify proteins with importance exceeding random noise [27,28]. To evaluate the robustness of AL-related proteins, we conducted a bootstrap stability analysis with 100 iterations. Proteins retained in at least 80% of bootstrap iterations were considered stable AL-associated proteins. Finally, SHAP analysis was applied to quantify and rank the contributions of retained proteins according to their mean absolute SHAP values.

Functional enrichment analyses were performed to characterize the potential biological functions and pathways of the identified AL-associated proteins. Specifically, Gene Ontology (GO) enrichment analysis was conducted to investigate associated biological processes, molecular functions, and cellular components. Kyoto Encyclopedia of Genes and Genomes (KEGG) pathway analysis was performed to further characterize the involvement of these proteins in known biological pathways. To investigate potential functional associations among the identified proteins, a protein–protein interaction (PPI) network was constructed using the Search Tool for the Retrieval of Interacting Genes/Proteins (STRING) database [23]. Interactions were retained using a high-confidence threshold, defined as a combined interaction score ≥ 0.700, to enhance the biological reliability of the network. Network-level connectivity was assessed using the STRING-reported PPI enrichment p-value, with p < 1.0 × 10^−6^ indicating that the observed number of interactions was significantly greater than expected by chance. Functional enrichment analyses were further evaluated using false discovery rate (FDR)-adjusted p-values, with FDR < 0.05 considered statistically significant.

Mediation analyses were performed to explore whether individual AL-associated proteins mediated the observed association between AL and incident lung cancer. Each identified protein was assessed separately as a potential mediator. In each mediation model, AL was specified as the exposure, the protein level as the mediator, and incident lung cancer as the outcome. The mediator model estimated the association between AL and protein levels, whereas the outcome model estimated the association of AL and the protein mediator with lung cancer risk. Both models were adjusted for the same covariates used in the primary analysis. The total effect of AL on lung cancer risk was partitioned into the natural direct effect and the natural indirect effect through each protein mediator. The proportion mediated was calculated as the indirect effect divided by the total effect. Bootstrap resampling with 1,000 replicates was used to estimate 95% confidence intervals for the indirect effect and proportion mediated. Multiple testing across proteins was controlled using FDR correction, and proteins with FDR < 0.05 were considered to have evidence of mediation. We subsequently performed subtype-specific mediation analyses. Given the limited number of events within individual lung cancer subtypes, these analyses were considered exploratory.

All statistical analyses were performed by R 4.5.1. All *P* values were 2-sided, and *P* < 0.05 was considered statistically significant.

## 3. Results

### 3.1 Baseline characteristics of study populations

A total of 336,945 participants for lung cancer incidence analysis (female: 53.6%, mean age 56.52 years old) were included. Baseline characteristics were presented in Table 1. Compared with the general study population, lung cancer patients tended to be older, male, former or current smokers, daily alcohol consumers, socioeconomically deprived, and more likely to have a family history of lung cancer.

**Table 1:** Baseline characteristics of UK Biobank participants.

Characteristics	No LC	LC Individuals	*p* value
	(n = 333956)	(n = 2989)	
Ages, mean (SD), years	56.48 (8.06)	61.83 (5.70)	< 0.001
Sex, n (%)			< 0.001
Female	179075 (53.6)	1399 (46.8)	
Male	154881 (46.4)	1590 (53.2)	
BMI, mean (SD), kg/m^2^	27.39 (4.74)	27.57 (4.74)	0.034
Ethnic, n (%)			0.005
Non-white	4814 (1.4)	22 (0.7)	
White	327908 (98.2)	2956 (98.9)	
Smoking status, n (%)			< 0.001
Never	180916 (54.2)	384 (12.8)	
Former	117205 (35.1)	1402 (46.9)	
Current	34423 (10.3)	1175 (39.3)	
Alcohol intake frequency, n (%)			< 0.001
Daily or almost daily	70523 (21.1)	775 (25.9)	
Three or four times a week	79759 (23.9)	571 (19.1)	
Once or twice a week	87313 (26.1)	669 (22.4)	
One to three times a month	37255 (11.2)	278 (9.3)	
Special occasions only	35802 (10.7)	390 (13.0)	
Never	22820 (6.8)	299 (10.0)	
Townsend, mean (SD)	-1.5(3.0)	-0.2 (3.5)	< 0.001
Education history, n (%)			< 0.001
College or university degree	223103 (66.8)	2479 (82.9)	
Others	107601 (32.2)	459 (15.4)	
IPAQ activity group, n (%)			< 0.001
Low	50520 (15.1)	518 (17.3)	
Moderate	110465 (33.1)	910 (30.4)	
High	110187 (33.0)	890 (29.8)	
Family history of lung cancer, n (%)			<0.001
No	290796 (87.1)	2313 (77.4)	
Yes	43160 (12.9)	676 (22.6)	
Follow-up time, median (IQR), years	13.72 (1.37)	7.23 (5.86)	<0.001
PRS for lung cancer, n (%)			<0.001
Q1	83489 (25.0)	621 (20.8)	
Q2-Q3	166978 (50.0)	1516 (50.7)	
Q4	83489 (25.0)	852 (28.5)	

The “Unknown” group of the categorical variables is not displayed. Bold values indicate the statistical significance P ≤ 0.05.

Abbreviations: LC, lung cancer; SD, standard deviation; BMI, Body Mass Index; Townsend, indicates Townsend deprivation index; IPAQ, indicates International Physical Activity Questionnaire; PRS, polygenic risk score.

The distribution of individual biomarkers and AL scores was presented in S2 Table. As a continuous variable, the mean AL score of lung cancer patients was 3.86, significantly higher than the 3.04 observed in the cancer-free population (*P* < 0.001). When categorizing AL into ternary distribution, statistical differences were discernible across the strata.

### 3.2 Allostatic load and incidence risk of lung cancer

In the UKB cohort, 2,989 participants developed lung cancer during a median follow-up of 13.79 years (IQR, 12.48–15.10 years). Higher AL was consistently associated with increased lung cancer risk (Table 2). In the continuous model, each one-unit increment in AL corresponded to a 7.3% higher risk (HR, 1.073; 95% CI, 1.053–1.094; *P* < 0.001). A graded association was observed in the categorical analysis. Compared with the normal group, both low (HR, 1.284; 95% CI, 1.065–1.547; *P* < 0.001) and high AL group (HR, 1.629; 95% CI, 1.347–1.971; *P* < 0.001) exhibited significantly elevated risks. Furthermore, risk trajectories differed substantially across AL strata, with participants in the high AL group experiencing significantly greater lung cancer risk than those in the low or normal groups (Fig 1).

**Table 2:** Association between AL and lung cancer incidence.

AL Category	Person-year Incidence (per 1,000 PY, 95% CI) of LC Cases	HR	95% CI	*p*-value
AL-Continuous	0.662 (0.639, 0.686)	1.073	(1.053, 1.094)	<0.001
AL-Ternary				
Normal	0.240 (0.200, 0.286)	Ref		
Low	0.540 (0.510, 0.570)	1.284	(1.065, 1.547)	0.009
High	0.949 (0.903, 0.996)	1.629	(1.347, 1.971)	<0.001

The model was adjusted for age, sex, body mass index, smoking status, alcohol intake frequency, International Physical Activity Questionnaire, education level, Townsend deprivation index and family history of lung cancer.

Abbreviations: AL, allostatic load; PY, person-year; HR, hazard ratio; CI, confidence interval.

**Fig 1:**
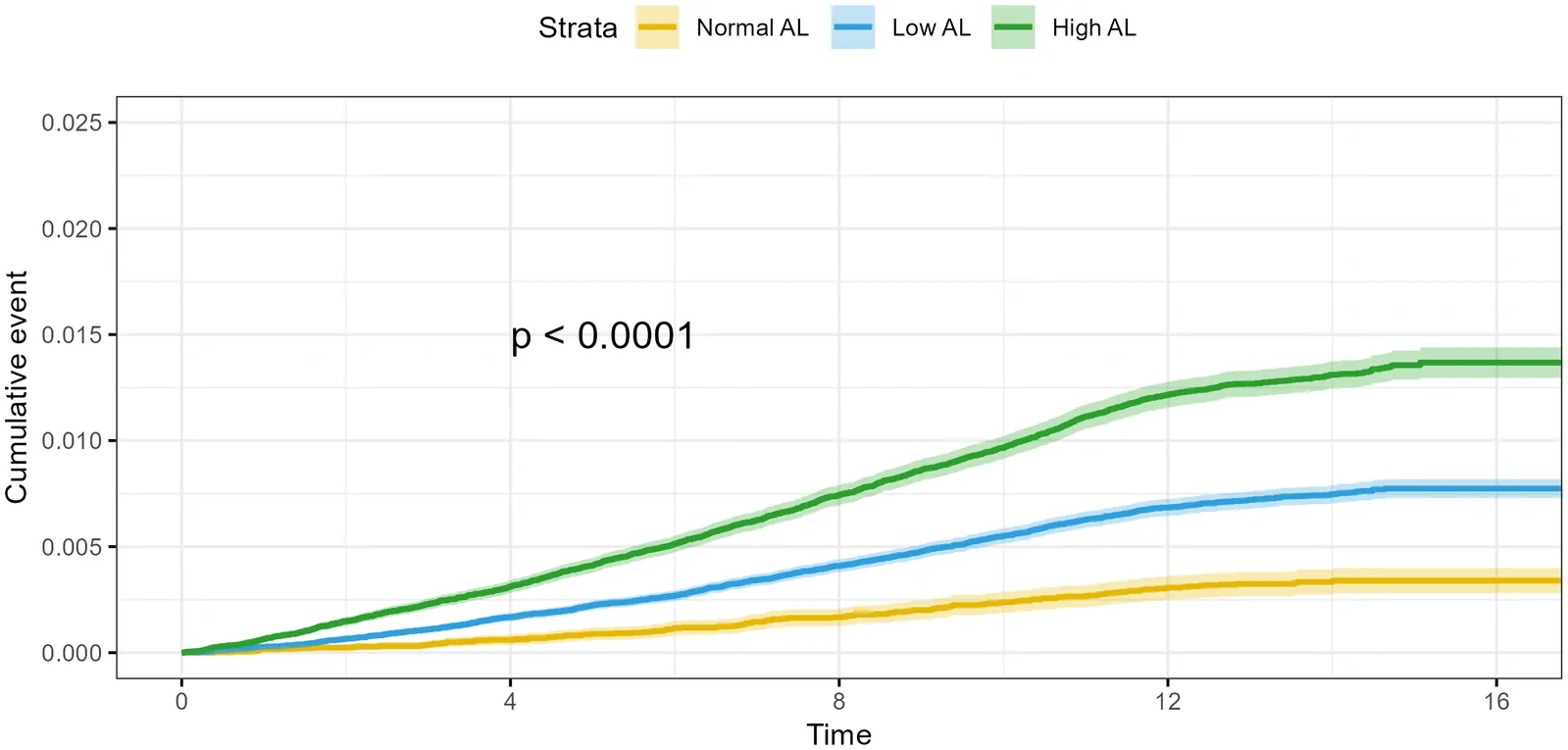
Cumulative risk of lung cancer by AL groups. Shaded regions represent the 95% confidence intervals. Abbreviations: AL, allostatic load.

The potential non-linear nature of the association was evaluated using an RCS analysis. The spline curve revealed a non-linear increasing trend (*P* = 0.001), with lung cancer risk remaining relatively stable at lower AL levels but rising significantly beyond a score of 3 (S2 Fig), suggesting a potential threshold effect above which the risk increases more rapidly.

To further delineate the contribution of individual physiological subsystems, we decomposed AL into its major subsystems and observed heterogeneous associations across systems (S3 Table). The inflammatory domain showed the strongest association, with a 30.1% higher risk per unit increase in score (HR = 1.301, 95% CI: 1.235–1.370; *P* < 0.001). Cardiovascular and metabolic domains also exhibited modest but statistically significant associations with increased lung cancer risk (HR = 1.054 and 1.057, respectively). In contrast, no significant associations were observed for the kidney domain (HR = 0.926, 95% CI: 0.846–1.013; *P* = 0.093).

### 3.3 Association of allostatic load with lung cancer histological subtypes

We observed heterogeneity across histological subtypes, with the strongest associations for SCLC (Table 3). Each one-unit AL increase conferred an 11.6% higher risk (HR = 1.116, 95% CI: 1.047–1.190; *P* < 0.001). In categorical analyses, high AL yielded over threefold greater risk compared with normal AL (HR = 3.275, 95% CI 1.316–8.150; *P* = 0.011), while low AL showed a borderline elevation (HR = 2.380, 95% CI 0.963–5.884; *P* = 0.060). Associations were weaker for NSCLC subtypes. In LUAD, a modest 5.0% per-unit increase was observed (HR = 1.050, 95% CI 1.019–1.082; *P* = 0.001), with significance confined to high AL (HR = 1.320, 95% CI 1.021–1.706; *P* = 0.034). LUSC showed an 8.3% per-unit increase (HR = 1.083, 95% CI 1.044–1.124; *P* < 0.001), with elevated risks for low (HR = 1.658, 95% CI 1.061–2.591; *P* = 0.026) and high AL (HR = 2.257, 95% CI 1.438–3.541; *P* < 0.001). No significant associations were detected for LCC, possibly due to limited number of cases.

**Table 3:** Association between AL and the risk of lung cancer subtypes.

	Person-year Incidence (per 1,000 PY, 95% CI) of Cases	Hazard Ratio	95% CI	*p value*
LUAD				
AL-Continuous	0.283 (0.268, 0.299)	1.050	(1.019, 1.082)	0.001
AL-Ternary				
Normal	0.140 (0.110, 0.177)	Ref		
Low	0.243 (0.223, 0.264)	1.077	(0.840, 1.381)	0.56
High	0.378 (0.350, 0.408)	1.320	(1.021, 1.706)	0.034
LUSC				
AL-Continuous	0.175 (0.163, 0.188)	1.083	(1.044, 1.124)	<0.001
AL-Ternary				
Normal	0.040 (0.025, 0.062)	Ref		
Low	0.131 (0.117, 0.147)	1.658	(1.061, 2.591)	0.026
High	0.273 (0.249, 0.299)	2.257	(1.438, 3.541)	<0.001
SCLC				
AL-Continuous	0.055 (0.049, 0.063)	1.116	(1.047, 1.190)	<0.001
AL-Ternary				
Normal	0.010 (0.003, 0.022)	Ref		
Low	0.042 (0.034, 0.051)	2.380	(0.963, 5.884)	0.060
High	0.088 (0.074, 0.103)	3.275	(1.316, 8.150)	0.011
LCC				
AL-Continuous	0.007 (0.004, 0.010)	1.077	(0.894, 1.298)	0.433
AL-Ternary				
Normal	0.002 (0.000, 0.011)	Ref		
Low	0.006 (0.003, 0.010)	1.786	(0.229, 13.870)	0.579
High	0.009 (0.005, 0.015)	1.836	(0.226, 14.927)	0.570

The model was adjusted for age, sex, body mass index, smoking status, alcohol intake frequency, indicates International Physical Activity Questionnaire, education level, Townsend deprivation index and family history of lung cancer.

Abbreviations: AL, allostatic load; LUAD, Lung Adenocarcinoma; LUSC, Lung Squamous Cell Carcinoma; SCLC, Small Cell Lung Cancer; LCC, Large Cell Carcinoma; PY, person-year; HR, hazard ratio; CI, confidence interval.

### 3.4 Joint effect and interaction of genetic factor and allostatic load on lung cancer

The construction of the PRS was based on 128 SNPs as detailed in S1 Table. Notably, participants with elevated PRSs demonstrated significantly higher cumulative risks of lung cancer incidence (*P* < 0.001). A 37.0% higher risk of lung cancer was observed for individuals in the highest PRS quartile versus those in the lowest (HR 1.370, 95% CI 1.235–1.519; *P* < 0.001), with a graded increase also observed for the intermediate group (HR 1.215, 95% CI 1.107–1.334), demonstrating a clear dose–response relationship (S3A Fig and S4 Table).

In joint analyses of genetic susceptibility and AL, both higher genetic risk and elevated AL were associated with increased lung cancer risk. Using the low genetic risk and normal AL group as the reference, hazards under normal AL did not differ significantly across genetic strata (all *P* > 0.050), and the low genetic risk with low AL group also showed no significant difference from the reference (HR = 1.262, 95% CI: 0.828–1.922; *P* = 0.279). In contrast, elevated AL was consistently associated with higher hazards across genetic strata, and the risk estimates increased from the low to the high genetic risk categories, with highest risk observed in the high genetic risk and high AL group (HR = 2.369, 95% CI: 1.565–3.583; *P* < 0.001) (Fig 2). Similar trends were observed in cumulative incidence analysis (S3B Fig). Groups with elevated AL exhibited accelerated accrual of lung cancer events, with the most pronounced accumulation occurring in the high genetic risk and high AL stratum, consistent with the joint analyses demonstrating progressively higher risks as both genetic susceptibility and AL increase.

**Fig 2:**
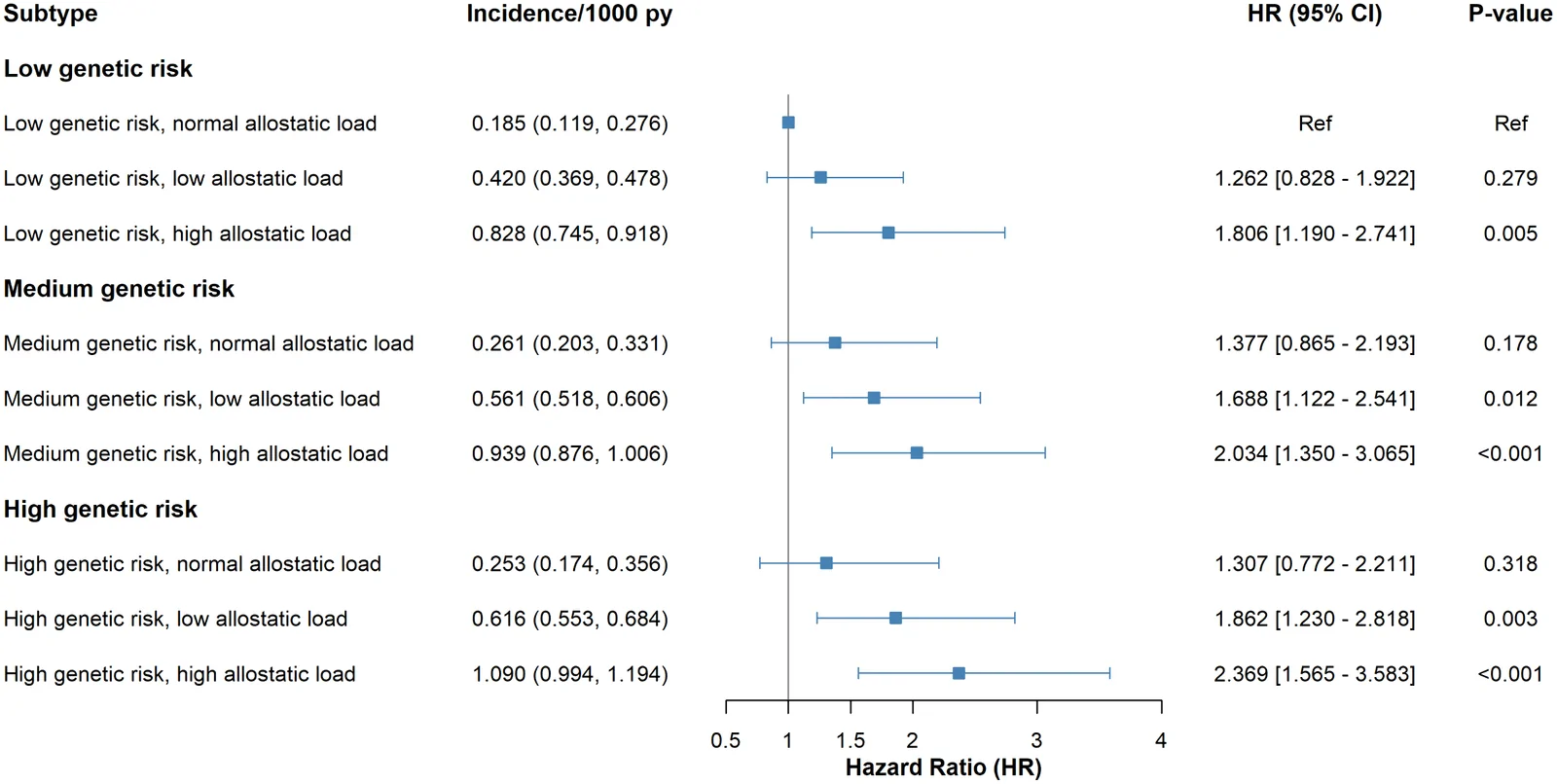
Risk of incident lung cancer according to allostatic load and genetic categories. The hazard ratios for lung cancer according to allostatic load and polygenic risk score categories were estimated by using Cox proportional hazard models with adjustment for age, sex, body mass index, smoking status, alcohol intake frequency, indicates International Physical Activity Questionnaire, education level, Townsend deprivation index and family history of lung cancer. Abbreviations: HR, hazard ratio; CI, confidence interval; PY, person-year.

We further assessed whether genetic susceptibility modified the association between AL and lung cancer risk on multiplicative and additive scales. The interaction term did not provide evidence that the association between AL and lung cancer risk differed across genetic risk strata (S5 Table), suggesting that the observed joint risk pattern mainly reflected cumulative risk contributions rather than departure from multiplicativity. Consistently, no evidence of additive interaction was observed, as the 95% CIs for RERI and AP included 0, and the 95% CI for SI included 1 (S6 Table).

### 3.5 AL and LDCT screening eligibility

To explore the potential relevance of AL for lung cancer screening risk stratification, we evaluated lung cancer risk across joint categories of AL and established LDCT screening eligibility criteria, including the USPSTF 2021 and NLST criteria (S7 and S8 Tables). Under the USPSTF 2021 criteria, continuous AL score was significantly associated with lung cancer risk among both screening-ineligible individuals (HR = 1.049, 95% CI: 1.022–1.078, P < 0.001) and screening-eligible individuals (HR = 1.045, 95% CI: 1.020–1.069, P < 0.001). In the joint analysis, lung cancer incidence increased across AL categories within both USPSTF eligibility groups, with the highest risk observed among USPSTF-eligible individuals with high AL (HR = 3.399, 95% CI: 2.765–4.177, P < 0.001).

Similar patterns were observed when applying the NLST criteria. Notably, high AL was significantly associated with increased lung cancer risk even among NLST-ineligible individuals (HR = 1.330, 95% CI: 1.102–1.604, P = 0.003), suggesting that AL may help identify individuals with elevated risk who are not captured by rule-based screening criteria. The highest risk was observed among NLST-eligible individuals with high AL (HR = 3.414, 95% CI: 2.804–4.158, P < 0.001).

We further evaluated whether adding AL improved model discrimination (S9 Table). Adding AL improved the discrimination of both screening eligibility models, with the C-index increasing from 0.723 to 0.755 for the USPSTF 2021 model (P < 0.001) and from 0.677 to 0.721 for the NLST model (P < 0.001). In contrast, the incremental improvement was modest when AL was added to the multivariable clinical model, with the C-index increasing from 0.836 to 0.839 (P < 0.001).

### 3.6 Subgroup and sensitivity analysis

We found no evidence of effect modification by age, BMI, ethnicity, alcohol intake frequency, physical activity, TDI, education, or family history of lung cancer (all *P* for interaction >0.050; S4 Fig and S10 Table). In contrast, we identified notable modifications by sex and smoking status. Compared to individuals with normal AL levels, the association between elevated AL and lung cancer risk varied substantially across these subgroups (both *P* for interaction < 0.050). Specifically, the link was stronger among men (HR = 1.905, 95% CI: 1.309–2.772) than among women (HR = 1.489, 95% CI: 1.186–1.871) in high AL level. Likewise, the relationship was most pronounced in current smokers (HR = 2.448, 95% CI: 1.681–3.564), followed by former smokers (HR = 1.673, 95% CI: 1.251–2.237), with no significant association evident among never smokers (HR = 0.882, 95% CI: 0.608–1.279). Further multiplicative interaction analyses yielded consistent findings, showing significant AL–smoking status interactions among former and current smokers compared with never smokers (S11 Table). The strongest interaction was observed between high AL and current smokers, with an interaction HR of 2.982 (95% CI: 1.810–4.913, P < 0.001), suggesting that individuals with both current smoking and high AL may represent a subgroup with particularly elevated lung cancer risk.

In sensitivity analyses with further adjustment for smoking-related covariates, including smoking duration, number of cigarettes smoked per day, and years since quitting smoking, the association between AL and lung cancer was slightly attenuated (S12 Table). The association was no longer significant for low AL (HR = 1.186, 95% CI: 0.970–1.451, P = 0.097), whereas it remained significant for continuous AL score (HR = 1.039, 95% CI: 1.018–1.061, P < 0.001) and high AL (HR = 1.370, 95% CI: 1.114–1.684, P = 0.003). Similar attenuation was observed in subtype-specific analyses (S13 Table). The association with LUAD was no longer evident after additional smoking adjustment. In contrast, LUSC remained associated with both continuous and categorical AL after additional smoking adjustment. For SCLC, although the estimates were attenuated compared with the main analysis, the associations persisted for continuous AL (HR = 1.081, 95% CI: 1.009–1.158; P = 0.027) and high AL (HR = 2.471, 95% CI: 1.056–5.782; P = 0.037). Additional sensitivity analyses further supported the robustness of the findings. The HRs for lung cancer remained stable after excluding participants with baseline COPD (S14 Table). The observed associations between AL and lung cancer risk did not materially change after excluding individuals diagnosed with lung cancer within the first year of follow-up or those who died within the first three years (S15 and S16 Tables). Finally, given the potential bias of conventional Cox regression in analyses with few events, Firth’s penalized Cox regression did not materially alter the associations between AL and lung cancer subtypes (S17 Table).

### 3.7 Identifying proteins associated with allostatic load

Using a machine learning–based feature selection framework, 111 AL-related proteins were identified. SHAP-based interpretation revealed distinct contributions of individual proteins, with the top 20 proteins ranked by mean absolute SHAP values (Fig 3A). Among these, LDLR, PLA2G7, and PRAP1 emerged as the most influential proteins, with higher expression levels associated with higher AL scores. These proteins have been implicated in lipid metabolism, inflammatory responses, and tissue remodeling, highlighting the relevance of metabolic and inflammatory processes to AL-related proteomic patterns. Conversely, lower expression levels of IGFBP3, PON3, and LPL were associated with higher SHAP values, consistent with inverse associations between these protein features and AL-related model predictions. These proteins are involved in endocrine regulation, antioxidant defense, and lipid homeostasis, although the biological implications of these inverse patterns require further investigation (Fig 3B).

**Fig 3:**
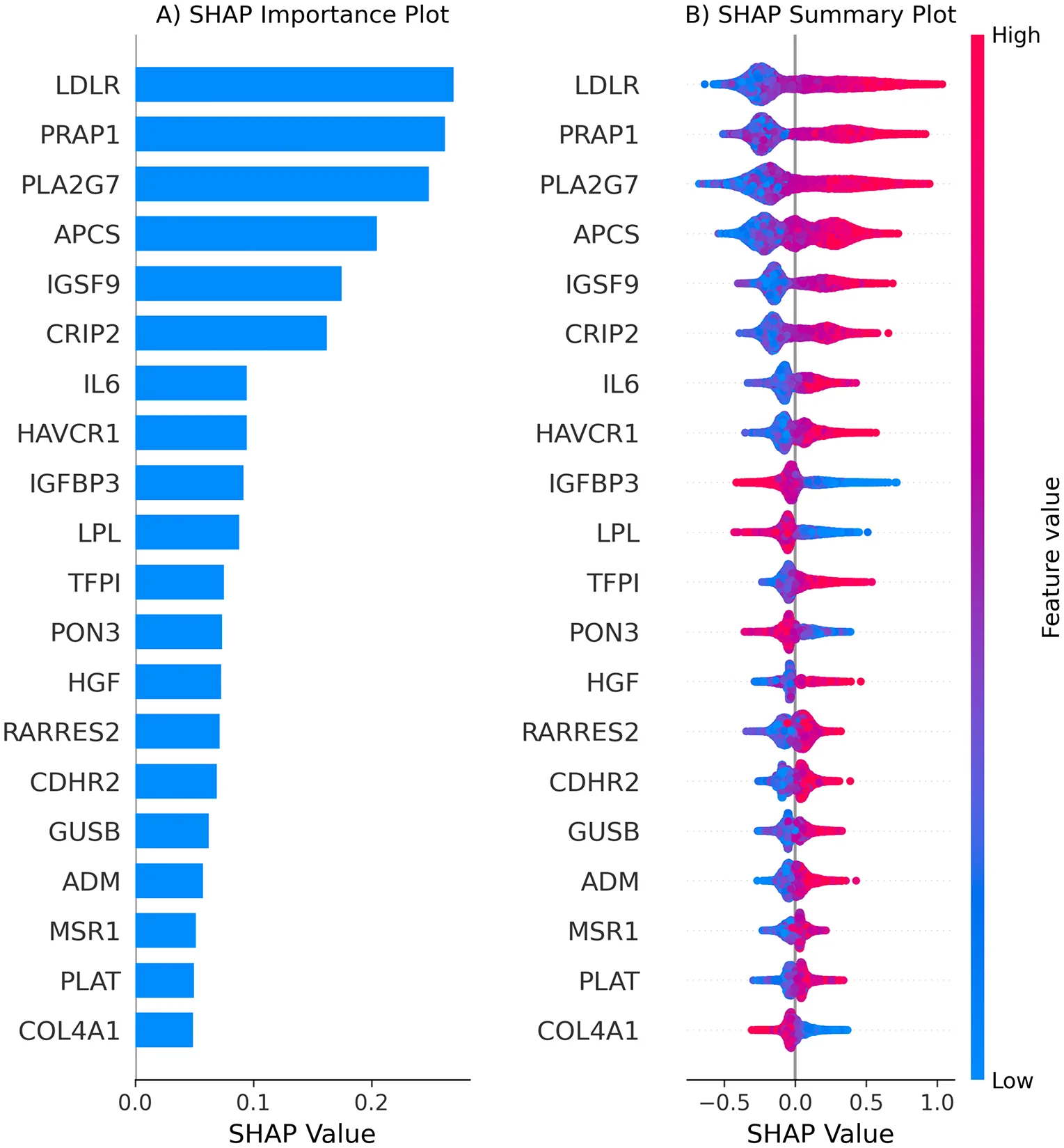
Top 20 most important proteins for predicting allostatic load. **(A)** SHAP importance plot of plasma proteins associated with allostatic load. **(B)** SHAP summary plot of plasma proteins associated with allostatic load. The length of each bar in the SHAP importance plot represents the magnitude of the feature’s impact, calculated by taking the mean of the absolute SHAP values across all participants for that protein, with longer bars indicating higher importance. The x-axis of the SHAP summary plot shows the SHAP values from the XGBoost model for each protein, with the SHAP value for each individual participant as an individual dot. The sign of SHAP values represents the effect on the model outcome. Dots are color coded according to the value of the protein feature.

### 3.8 Enrichment analysis and Protein-Protein Interaction analysis

To characterize the biological functions and molecular pathways associated with the proteins linked to AL, we performed functional enrichment analyses. Complementary GO enrichment analysis indicated overrepresentation of biological processes related to plasma lipoprotein particle clearance and remodeling, as well as regulation of the MAPK cascade, together with molecular functions such as receptor ligand activity and lipoprotein binding (S5A–S5C Fig). KEGG pathway analysis further revealed significant enrichment in cholesterol metabolism, cytokine–cytokine receptor interaction, PI3K–Akt signaling, and MAPK signaling pathways (S5D Fig).

PPI analysis using the maximal clique centrality (MCC) method identified several top-ranking nodes, including APOA1, PLTP and APOC1, which are pivotal in lipid metabolism and transport. Additionally, PLA2G7, IL6, and APOM were prominent, underscoring their strong ties to inflammatory response and immune regulation. Other key hub proteins, such as KITLG, TTR, HGF, and LPL, were associated with hematopoiesis, endocrine function, tissue regeneration, and triglyceride metabolism, respectively (S5E Fig).

### 3.9 Mediation analysis of proteins

The associations of 111 identified proteins with the risk of lung cancer were shown in S18 Table. A total of 34 proteins showed supported mediation signals for the association between AL and LC risk (FDR < 0.05). HGF showed the largest estimated proportion mediated, accounting for 28% of the estimated total AL-LC association (95% CI: 13.5%-72.5%, p < 0.001). RARRES2 also emerged as a significant mediator, contributing 23% to the total effect (95% CI: 5.2%-60.6%, p < 0.001), while GDF15 mediated 22% (95% CI: 13.4%-60.2%, p < 0.001).

In the subtype-specific mediation analysis, 39 significant protein–subtype mediation signals were identified, including 15 for LUAD, 19 for SCC, and 5 for SCLC (S19 Table). A common set of nine proteins showed significant indirect-effect signals for both LUAD and SCC, including HGF, IL6, GDF15, ADM, BCAN, NTRK3, NCAN, ACAN, and MSTN, consistent with partially shared protein-related pathways across NSCLC subtypes. For SCLC, five proteins showed significant indirect effects, including GDF15, LECT2, KITLG, BCAN, and CDH2. Among these, GDF15, LECT2, KITLG, and BCAN showed positive indirect-effect patterns, with estimated mediated proportions of 61.0%, 30.3%, 30.8%, and 10.4%, respectively. CDH2 showed an inverse indirect effect, suggesting a potentially opposing or compensatory pathway.

## 4. Discussion

Leveraging a large prospective cohort, our study provides robust evidence that elevated AL is associated with an increased incidence of lung cancer. The association showed a graded pattern and varied across physiological subsystems, with the inflammatory domain showing the strongest association, followed by the cardiovascular and metabolic domains, whereas no significant association was observed for the kidney domain. Subtype-specific analyses further demonstrated significant associations of AL with LUAD, LUSC, and SCLC, whereas no clear evidence of association was detected for LCC. Joint analyses demonstrated that elevated AL and genetic predisposition independently contributed to lung cancer risk, with no additive interaction detected. Beyond etiological associations, AL also demonstrated potential relevance for LDCT screening-oriented risk stratification by further differentiating lung cancer risk within USPSTF 2021 and NLST eligibility groups and improving the discrimination of rule-based screening eligibility models. In addition, integrative plasma proteomic analyses identified 111 AL-associated proteins, of which 34 showed mediation signals for the AL–lung cancer association, including prominent signals for HGF, RARRES2, and GDF15.

AL reflects the cumulative physiological burden of chronic stress and has been increasingly linked to adverse health outcomes, including cancer [13,29]. Our findings extend previous UKB evidence by demonstrating that elevated AL is associated not only with overall lung cancer risk but also with major histological subtypes. Although the inflammatory subsystem showed the strongest association, cardiovascular and metabolic subsystems also contributed to lung cancer risk, suggesting that AL-related risk may reflect the combined influence of chronic inflammation, cardiometabolic dysfunction, and broader multisystem vulnerability. Previous studies have suggested that sustained physiological stress may be accompanied by systemic inflammatory responses, tissue injury, and altered immune surveillance, processes that have also been implicated in tumor development [30,31]. In parallel, cardiovascular dysfunction and metabolic dysregulation may further exacerbate these effects through impaired tissue perfusion, local hypoxia, and activation of pro-proliferative signaling pathways [32–34], collectively suggesting that AL is not merely a stress marker but an integrated indicator of multisystem physiological burden that may contribute to lung cancer development.

Notably, the strongest association between AL and lung cancer was observed for SCLC, with comparatively weaker associations for LUAD and LUSC. Compared with LUAD, which has a stronger contribution from inherited susceptibility and non-smoking-related pathways, SCLC is more closely linked to heavy smoking exposure and smoking-related molecular injury [35]. SCLC is characterized by rapid proliferation and a high mutational burden, and inflammatory and oxidative stress-related processes have been implicated in its pathobiology [36]. After further adjustment for smoking-related variables, the association with LUAD was attenuated, whereas associations for LUSC and SCLC persisted for key AL indicators. Given that smoking burden has been associated with elevated AL, our findings suggest that AL may partly capture smoking-related physiological burden while also reflecting broader systemic vulnerability beyond conventional smoking metrics [13,37]. This may be especially relevant to SCLC, in which smoking-related molecular injury, inflammation, and oxidative stress are closely intertwined. Individuals with high AL should therefore be encouraged to adopt smoking cessation, maintain healthy lifestyle habits, and seek appropriate medical care to mitigate systemic stress and reduce cancer risk.

We further observed that elevated AL and higher PRS were independently associated with increased lung cancer risk, with individuals exhibiting both factors having the highest risk. However, neither multiplicative nor additive interaction provided evidence that genetic susceptibility modified the AL–lung cancer association, suggesting that the joint pattern is better interpreted as cumulative risk contribution rather than biological synergy. The absence of interaction also suggests that AL and genetic susceptibility may reflect partially distinct dimensions of risk, although both may be related to lung cancer risk. The observed cumulative risk pattern highlights the potential value of integrating systemic stress markers with genetic risk information to improve lung cancer risk stratification.

Beyond genetic risk stratification, our findings also suggest that AL may have potential relevance for risk-adapted lung cancer screening. Current LDCT screening eligibility criteria, including USPSTF 2021 and NLST, are rule-based and largely driven by age and smoking exposure [25,26]. Although these criteria are practical and widely used, they may not fully capture multi-system physiological vulnerability. By jointly evaluating AL with established LDCT screening eligibility criteria, we observed that AL further differentiated lung cancer risk within both screening-eligible and screening-ineligible populations. High AL was associated with elevated lung cancer risk among NLST-ineligible individuals, suggesting that AL may provide additional risk information for individuals not selected by conventional rule-based criteria. The highest risks were consistently observed among screening-eligible individuals with high AL, indicating that AL may also help refine risk prioritization within populations already meeting LDCT screening criteria. From a digital health perspective, AL could be incorporated into electronic health records or digital risk assessment platforms as it can be derived from routinely collected clinical biomarkers. With further external validation, such integration could support scalable risk stratification and potentially identify physiologically vulnerable individuals whose risk is not fully captured by conventional rule-based LDCT eligibility criteria.

To explore potential biological correlates, we integrated plasma proteomics with machine learning-based feature selection. We identified 111 plasma proteins associated with AL, with enrichment analyses highlighting inflammatory and immune processes, lipid and energy metabolism, and growth factor-related signaling pathways. Of note, dysregulated lipid metabolism and chronic inflammation are established hallmarks of lung tumor development [38–40]. Mediation analyses further identified 34 proteins showing mediation signals for the association between AL and lung cancer risk, providing exploratory biological context for the observed epidemiological association. Among these, HGF, RARRES2, and GDF15 emerged as the most influential mediators. Subtype-specific mediation analysis further highlighted GDF15 as a consistent putative mediator, with a particularly interpretable mediation pattern observed for SCLC, suggesting that GDF15 may represent a shared protein marker connecting systemic physiological dysregulation with lung cancer risk, while also reflecting subtype-specific vulnerability.

Examining these key protein mediators provides biological context for the observed association between AL and lung cancer risk. HGF is a potent growth factor whose aberrant activation of the c-Met receptor pathway drives epithelial cell proliferation, migration, invasion, and angiogenesis [41,42]. Elevated HGF signaling in chronically stressed individuals could thus directly promote tumor initiation and progression by enhancing these malignant capabilities. RARRES2 is an adipokine that operates at the intersection of inflammatory and metabolic networks [43]. Previous studies have implicated RARRES2 in tumor-related angiogenesis and cancer cell invasiveness, providing additional context for its association in the present analysis. GDF15, a stress-responsive cytokine, exemplifies the immunosuppressive arm of AL’s impact. GDF15 possesses anti-inflammatory properties and helps tumor cells evade immune surveillance by macrophages in early tumorigenesis [44]. Elevated GDF15 under chronic stress might blunt the body’s anti-tumor immune response and enable incipient tumors to establish and grow [34]. Collectively, these protein findings point to inflammatory, metabolic, growth factor, and immune-related processes that may provide biological context for the association between AL and lung cancer risk. However, given the observational design and the exploratory nature of the proteomic and mediation analyses, these findings should be considered hypothesis-generating rather than evidence of a causal biological mechanism.

This study has several strengths. A key strength of this study is that it strengthens existing evidence by further exploring the relationship between AL and the incidence of lung cancer subtypes, while simultaneously exploring potential protein mediators underlying the relationship. In addition, it is based on a large-scale prospective cohort with a rigorous study design and a substantial sample size. A variety of sensitivity analyses further ensured the robustness of our findings. Moreover, we evaluated the contribution of genetic factors to the association of AL and lung cancer, enabling us to precisely determine the effects of AL across groups with different levels of genetic susceptibility. Furthermore, we evaluated the potential relevance of AL for LDCT screening-oriented risk stratification by combining AL categories with established USPSTF 2021 and NLST eligibility criteria. Our analysis demonstrated that AL could further differentiate risk within both screening-eligible and screening-ineligible populations, highlighting its potential value as a clinically accessible and digitally implementable risk stratification marker. However, the present study has several limitations. First, our study population consisted predominantly of individuals of European ancestry, which may limit the generalizability of our findings. Future research should include participants from more diverse ancestry groups. Second, AL biomarkers were measured only at baseline, which may not fully capture their long-term variation during follow-up. Third, although we adjusted for a wide range of potential confounders, the influence of residual or unmeasured confounding cannot be entirely excluded. Fourth, given the limited number of subtype-specific events, bootstrap estimates of mediated proportions may be unstable. The subtype-specific mediation findings should be interpreted as exploratory.

## 5. Conclusion

The results of this cohort study provided compelling evidence that elevated AL was associated with an increased risk of lung cancer, with particularly pronounced associations among SCLC. AL also complemented genetic susceptibility and established LDCT screening eligibility criteria in identifying individuals at higher lung cancer risk, suggesting its potential value as a clinically accessible marker for risk-adapted screening. These findings position AL as a multidimensional marker of multisystem physiological vulnerability with potential utility for complementing conventional genetic and screening-based approaches to lung cancer risk stratification.

## Supporting information

S1 Fig Workflow of association between allostatic load and lung cancer incidence. (TIF)

S2 Fig Association of AL with lung cancer risk across different AL scores. (TIF)

S3 Fig Cumulative incidence of lung cancer stratified by A) genetic risk and B) joint categories of genetic risk and allostatic load. (TIF)

S4 Fig Subgroup analyses of the associations between AL (three-categories, normal as reference) and the risk of lung cancer. (TIF)

S5 Fig Functional enrichment and protein–protein interaction network of AL-associated proteins. (TIF)

S1 Table Summary results of 128 SNPs used for polygenic risk score in the study of Hung RJ et al. (DOCX)

S2 Table Distribution of individual biomarkers and AL score category among UK Biobank cohort. (DOCX)

S3 Table Association between AL subsystems and the risk of lung cancer. (DOCX)

S4 Table Association of genetic risk levels with risk of lung cancer incidence. (DOCX)

S5 Table Interaction between AL and genetic susceptibility in relation to lung cancer incidence. (DOCX)

S6 Table RERI, AP and SI for additive interaction between AL and genetic categories. (DOCX)

S7 Table Lung cancer risk according to USPSTF 2021 eligibility and AL category. (DOCX)

S8 Table Lung cancer risk according to NLST eligibility and AL category. (DOCX)

S9 Table Incremental discrimination after adding AL to LDCT screening eligibility models. (DOCX)

S10 Table Subgroups analyses of the associations between AL (three-categories, normal as reference) and the risk of lung cancer. (DOCX)

S11 Table Interaction between AL and smoking status in relation to lung cancer incidence. (DOCX)

S12 Table Sensitivity analysis for association between AL and lung cancer incidence additionally adjusted for smoking related covariates. (DOCX)

S13 Table Sensitivity analysis for association between AL and lung cancer subtypes additionally adjusted for smoking related covariates. (DOCX)

S14 Table Sensitivity analysis for association between AL and lung cancer incidence excluding the population diagnosed with chronic obstructive pulmonary disease at enrollment. (DOCX)

S15 Table Sensitivity analysis for association between AL and lung cancer incidence excluding the population who developed lung cancer within the first year of enrollment. (DOCX)

S16 Table Sensitivity analysis for association between AL and lung cancer incidence excluding the population who died within three years of enrollment. (DOCX)

S17 Table Sensitivity analysis for association between AL and lung cancer Subtype Incidence using Firth’s Cox Regression. (DOCX)

S18 Table Association of AL with risk of lung cancer mediated by individual protein. (DOCX)

S19 Table Subtype-specific mediation analysis of protein mediators in NSCLC. (DOCX)

## Abbreviations

AL: Allostatic load
AP: Attributable Proportion due to the Interaction
BMI: Body Mass Index
CI: Confidence Interval
COPD: Chronic Obstructive Pulmonary Disease
Cre: Creatinine
CRP: C-reactive Protein
DBP: Diastolic Blood Pressure
GWAS: Genome-wide Association Studies
HbA1c: Glycated Hemoglobin
HDL: High-density Lipoprotein Cholesterol
HR: Hazard Ratio
ICD-10: International Classification of Diseases, 10th Revision
ICD-O-3: International Classification of Diseases for Oncology, Third Edition
IGF-1: Insulin-like Growth Factor-1
ILCCO: International Lung Cancer Consortium
IPAQ: International Physical Activity Questionnaire
IQR: Interquartile Range
LCC: Large Cell Carcinoma
LDCT: Low-Dose Computed Tomography
LDL: Low-density Lipoprotein Cholesterol
LUAD: Lung Adenocarcinoma
LUSC: Squamous Cell Lung Carcinoma
NSCLC: Non-small-cell Lung Cancer
NLST: National Lung Screening Trial
PR: Pulse Rate
PRS: Polygenic Risk Score
RERI: Relative Excess Risk due to the Interaction
SBP: Systolic Blood Pressure
SCLC: Small-cell Lung Cancer
SD: Standard Deviation
SI: Synergy Index
SNPs: Single-nucleotide Polymorphisms
TC: Total Cholesterol
TDI: Townsend Deprivation Index
TG: Triglycerides
UKB: UK Biobank
UKB PPP: UK Biobank Pharma Proteomics Project
USPSTF: United States Preventive Services Task Force
WHR: Waist-to-hip Ratio
XGBoost: eXtreme Gradient Boosting
