# Supplementary material for: Allostatic load, genetic susceptibility, and lung cancer risk: Evidence from a prospective cohort and integrative proteomic analyses
Source: PLOS Digit Health. 2026 Oct 14;5(10):e0001766. doi: 10.1371/journal.pdig.0001766 (PMC13667778; doi:10.1371/journal.pdig.0001766)
Supplement: Supplementary file 1 [file pdig.0001766.s006.docx]

**S1 Table** Summary results of 128 SNPs used for polygenic risk score in the study of Hung RJ et al.

| **rsID** | **chr** | **position** | **effect allele** | **other allele** | **EAF** | **Locus name** | **OR** | **Beta** |
| --- | --- | --- | --- | --- | --- | --- | --- | --- |
| rs71658797 | 1 | 77967507 | A | T | 0.1 | AK5 | 1.14 | 0.131028 |
| rs13080835 | 3 | 189357199 | T | G | 0.49 | TP63 | 0.94 | -0.06188 |
| rs7705526 | 5 | 1285974 | A | C | 0.34 | TERT | 1.12 | 0.113329 |
| rs112290073 | 5 | 1286032 | A | G | 0.01 | TERT | 1.39 | 0.329304 |
| rs2736098 | 5 | 1294086 | T | C | 0.28 | TERT | 1.14 | 0.131028 |
| rs2853668 | 5 | 1300025 | T | G | 0.24 | TERT | 1.08 | 0.076961 |
| rs401681 | 5 | 1322087 | T | C | 0.43 | CLPTM1L | 0.87 | -0.13926 |
| rs466502 | 5 | 1325767 | G | A | 0.44 | CLPTM1L | 0.91 | -0.09431 |
| rs6903823 | 6 | 28354519 | G | A | 0.2 | ZKSCAN3 | 1.07 | 0.067659 |
| rs116822326 | 6 | 31434111 | G | A | 0.16 |  | 1.15 | 0.139762 |
| rs2855812 | 6 | 31504943 | T | G | 0.22 | MICB | 1.05 | 0.04879 |
| rs805262 | 6 | 31628733 | T | C | 0.47 | C6orf47 | 1.07 | 0.067659 |
| rs6916278 | 6 | 31678774 | A | G | 0.05 | LY6G6F_LY6G6D | 0.9 | -0.10536 |
| rs3129763 | 6 | 32590925 | A | G | 0.22 |  | 1.12 | 0.113329 |
| rs114544105 | 6 | 32667852 | A | G | 0.2 | HLA_DQB1 | 1.06 | 0.058269 |
| rs6920364 | 6 | 167376466 | C | G | 0.46 |  | 1.07 | 0.067659 |
| rs11780471 | 8 | 27344719 | A | G | 0.06 | EPHX2 | 0.87 | -0.13926 |
| rs4236709 | 8 | 32410110 | G | A | 0.22 | NRG1 | 1.07 | 0.067659 |
| rs885518 | 9 | 21830157 | G | A | 0.1 | MTAP | 1.09 | 0.086178 |
| rs2007153 | 9 | 136503819 | T | C | 0.37 | DBH | 0.96 | -0.04082 |
| rs11591710 | 10 | 105687632 | C | A | 0.14 |  | 1.07 | 0.067659 |
| rs1056562 | 11 | 118125625 | T | C | 0.48 | MPZL2 | 1.07 | 0.067659 |
| rs7953330 | 12 | 998819 | C | G | 0.31 | WNK1 | 0.92 | -0.08338 |
| rs11571833 | 13 | 32972626 | T | A | 0.01 | BRCA2 | 1.6 | 0.470004 |
| rs689647 | 15 | 43762196 | T | C | 0.11 | TP53BP1 | 0.93 | -0.07257 |
| rs66759488 | 15 | 47577451 | A | G | 0.36 | SEMA6D | 1.07 | 0.067659 |
| rs77468143 | 15 | 49376624 | G | T | 0.25 |  | 0.92 | -0.08338 |
| rs3885951 | 15 | 78825917 | G | A | 0.11 | HYKK | 1.17 | 0.157004 |
| rs55781567 | 15 | 78857986 | G | C | 0.37 | CHRNA5 | 1.3 | 0.262364 |
| rs7177699 | 15 | 79089734 | C | T | 0.44 | ADAMTS7 | 1.13 | 0.122218 |
| rs62070270 | 17 | 29936962 | G | A | 0.45 | EFCAB5 | 1.03 | 0.029559 |
| rs1542752 | 17 | 72938100 | T | C | 0.16 | OTOP3 | 1.04 | 0.039221 |
| rs56113850 | 19 | 41353107 | T | C | 0.44 | CYP2A6 | 0.88 | -0.12783 |
| rs41309931 | 20 | 62326579 | T | G | 0.12 | RTEL1 | 1.08 | 0.076961 |
| rs17879961 | 22 | 29121087 | G | A | 0.01 | CHEK2 | 0.6 | -0.51083 |
| rs71641333 | 1 | 78743005 | A | T | 0.06 | MGC27382 | 1.03 | 0.029559 |
| rs78062588 | 1 | 154566225 | C | T | 0.06 | ADAR | 0.95 | -0.05129 |
| rs114737056 | 1 | 168511081 | A | G | 0.12 | XCL2 | 0.95 | -0.05129 |
| rs145733018 | 2 | 38567201 | C | T | 0.02 | ATL2 | 1.04 | 0.039221 |
| rs79368540 | 2 | 45189737 | T | C | 0.15 |  | 1.05 | 0.04879 |
| rs11692700 | 2 | 67510377 | C | T | 0.03 | LINC01828 | 1.05 | 0.04879 |
| rs114928225 | 2 | 119449740 | A | T | 0.01 |  | 1.17 | 0.157004 |
| rs7592999 | 2 | 140398327 | C | T | 0.04 |  | 0.92 | -0.08338 |
| rs722864 | 2 | 173983204 | A | G | 0.19 | MAP3K20 | 0.99 | -0.01005 |
| rs1866631 | 2 | 174075761 | G | A | 0.4 | MAP3K20 | 0.99 | -0.01005 |
| rs185666783 | 4 | 67833774 | G | C | 0.29 | LOC105377262 | 1.06 | 0.058269 |
| rs7676823 | 4 | 164007992 | G | A | 0.34 |  | 0.98 | -0.0202 |
| rs78154696 | 5 | 1000156 | A | G | 0.03 |  | 1.09 | 0.086178 |
| rs112333466 | 5 | 1249816 | T | C | 0.01 | TERT | 1.07 | 0.067659 |
| rs56345976 | 5 | 1276873 | G | A | 0.42 | TERT | 1.02 | 0.019803 |
| rs2853677 | 5 | 1287194 | G | A | 0.42 | TERT | 1.06 | 0.058269 |
| rs112401627 | 5 | 1300269 | A | G | 0.03 |  | 1.05 | 0.04879 |
| rs6875416 | 5 | 90250631 | T | A | 0.21 | ADGRV1 | 0.96 | -0.04082 |
| rs114136906 | 5 | 150121458 | C | G | 0.02 | DCTN4 | 1.14 | 0.131028 |
| rs2316515 | 6 | 410848 | A | G | 0.41 | IRF4 | 0.96 | -0.04082 |
| rs629444 | 6 | 25885814 | T | C | 0.1 | HIST1H2APS2 | 1 | 0 |
| rs2179517 | 6 | 26198845 | C | G | 0.49 | HIST1H3D | 1 | 0 |
| rs68141011 | 6 | 28217797 | T | G | 0.13 | ZKSCAN4 | 0.99 | -0.01005 |
| rs114722608 | 6 | 29223493 | C | G | 0.09 | LOC101929006 | 0.79 | -0.23572 |
| rs115123779 | 6 | 29477821 | T | G | 0.18 | LOC105375009 | 1.01 | 0.00995 |
| rs138488080 | 6 | 29606761 | A | G | 0.15 | SUMO2P1 | 1 | 0 |
| rs114192654 | 6 | 29759750 | A | G | 0.34 |  | 0.98 | -0.0202 |
| rs116675020 | 6 | 29922740 | G | A | 0.38 | HLA_W | 0.99 | -0.01005 |
| rs115993819 | 6 | 30074163 | A | G | 0.24 | TRIM31 | 1 | 0 |
| rs116534499 | 6 | 30138162 | G | C | 0.43 | TRIM15 | 1.01 | 0.00995 |
| rs116629156 | 6 | 30864829 | C | T | 0.41 | DDR1 | 1 | 0 |
| rs114103504 | 6 | 31002452 | G | A | 0.49 | MUC22 | 0.98 | -0.0202 |
| rs114052224 | 6 | 31067852 | G | A | 0.48 |  | 1.02 | 0.019803 |
| rs2233959 | 6 | 31081065 | C | T | 0.41 | C6orf15 | 1.01 | 0.00995 |
| rs114689412 | 6 | 31117577 | G | C | 0.13 | CCHCR1 | 1 | 0 |
| rs2596499 | 6 | 31321429 | A | T | 0.3 | HLA_B | 0.99 | -0.01005 |
| rs2596496 | 6 | 31322782 | C | G | 0.36 | HLA_B | 0.99 | -0.01005 |
| rs2596490 | 6 | 31324996 | G | C | 0.23 |  | 1.01 | 0.00995 |
| rs115176861 | 6 | 31412961 | C | T | 0.48 | HCP5 | 1.03 | 0.029559 |
| rs553108 | 6 | 31840455 | A | G | 0.38 | SLC44A4 | 1 | 0 |
| rs115200960 | 6 | 32335204 | A | G | 0.17 | C6orf10 | 1 | 0 |
| rs12722051 | 6 | 32609147 | T | A | 0.18 | HLA_DQA1 | 0.98 | -0.0202 |
| rs116767258 | 6 | 32757737 | G | A | 0.39 |  | 0.96 | -0.04082 |
| rs7383287 | 6 | 32783086 | G | A | 0.2 | HLA_DOB | 1.03 | 0.029559 |
| rs117534741 | 6 | 72384541 | A | G | 0.02 |  | 1.17 | 0.157004 |
| rs1321817 | 6 | 117734267 | G | A | 0.37 | ROS1 | 0.99 | -0.01005 |
| rs6957511 | 7 | 130668618 | C | T | 0.4 | LINC_PINT | 1.02 | 0.019803 |
| rs2565064 | 8 | 27327841 | C | G | 0.29 | CHRNA2 | 1.03 | 0.029559 |
| rs67749759 | 8 | 27397087 | T | C | 0.07 | EPHX2 | 1.03 | 0.029559 |
| rs111960002 | 8 | 144722420 | C | T | 0.05 | ZNF623 | 1.04 | 0.039221 |
| rs10118776 | 9 | 6227418 | G | A | 0.06 | IL33 | 1.08 | 0.076961 |
| rs17185553 | 9 | 17934120 | C | G | 0.08 |  | 1.02 | 0.019803 |
| rs2518717 | 9 | 21959751 | C | T | 0.36 | RP11_145E5.5 | 1 | 0 |
| rs28557075 | 9 | 22066572 | A | G | 0.09 | CDKN2B_AS1 | 1.11 | 0.10436 |
| rs1333040 | 9 | 22083404 | C | T | 0.46 | CDKN2B_AS1 | 1 | 0 |
| rs4879704 | 9 | 33427322 | C | A | 0.33 |  | 0.98 | -0.0202 |
| rs191205566 | 9 | 102587233 | T | C | 0.02 | NR4A3 | 1.21 | 0.19062 |
| rs75685923 | 9 | 136275229 | T | C | 0.03 | REXO4 | 1.09 | 0.086178 |
| rs7897454 | 10 | 102011702 | A | G | 0.04 | CWF19L1 | 1.06 | 0.058269 |
| rs62621207 | 10 | 102672248 | T | A | 0.05 | SLF2 | 1.07 | 0.067659 |
| rs78853063 | 11 | 57250026 | T | C | 0.08 | SLC43A1 | 0.97 | -0.03046 |
| rs78334599 | 11 | 115998756 | A | G | 0.04 |  | 0.89 | -0.11653 |
| rs7487683 | 12 | 1036042 | T | C | 0.04 | RAD52 | 0.94 | -0.06188 |
| rs73351723 | 12 | 58831070 | A | G | 0.13 |  | 1 | 0 |
| rs9668978 | 12 | 64913237 | T | G | 0.29 | RP11_439H13.2 | 1.05 | 0.04879 |
| rs9602270 | 13 | 84281063 | T | A | 0.05 |  | 1.04 | 0.039221 |
| rs8003466 | 14 | 34013721 | A | G | 0.18 | NPAS3 | 0.96 | -0.04082 |
| rs8031813 | 15 | 49253961 | C | A | 0.31 | SHC4 | 0.99 | -0.01005 |
| rs6493361 | 15 | 49615952 | G | C | 0.34 | GALK2 | 1.02 | 0.019803 |
| rs11855650 | 15 | 70431773 | T | G | 0.38 |  | 1.06 | 0.058269 |
| rs79149102 | 15 | 75055819 | T | C | 0.03 |  | 1.09 | 0.086178 |
| rs2229961 | 15 | 78880752 | A | G | 0.02 | CHRNA5 | 1.05 | 0.04879 |
| rs8192479 | 15 | 78909398 | T | C | 0.02 | CHRNA3 | 1.08 | 0.076961 |
| rs2869551 | 15 | 78981423 | G | A | 0.01 | CHRNB4 | 0.91 | -0.09431 |
| rs12593207 | 15 | 78987225 | A | G | 0.1 | CHRNB4 | 1.01 | 0.00995 |
| rs189146505 | 15 | 79058730 | G | A | 0.04 | ADAMTS7 | 1 | 0 |
| rs28450923 | 15 | 79065557 | G | A | 0.13 | ADAMTS7 | 0.99 | -0.01005 |
| rs28624856 | 15 | 79075233 | C | T | 0.24 | ADAMTS7 | 1 | 0 |
| rs77719127 | 15 | 79110783 | T | C | 0.17 | MORF4L1 | 1 | 0 |
| rs76164573 | 15 | 79198760 | G | T | 0.04 |  | 0.97 | -0.03046 |
| rs78442819 | 16 | 10740982 | C | G | 0.19 | TEKT5 | 0.97 | -0.03046 |
| rs9926896 | 16 | 26980646 | C | T | 0.01 |  | 1.22 | 0.198851 |
| rs17181550 | 17 | 70299958 | G | T | 0.43 |  | 0.97 | -0.03046 |
| rs79421398 | 18 | 20741135 | C | T | 0.05 | CABLES1 | 1.04 | 0.039221 |
| rs66500423 | 19 | 41195170 | C | T | 0.3 | NUMBL | 1.01 | 0.00995 |
| rs4803356 | 19 | 41207206 | G | C | 0.07 | ADCK4 | 1 | 0 |
| rs11881918 | 19 | 41334199 | A | G | 0.09 | CTC_490E21.12 | 0.93 | -0.07257 |
| rs2258380 | 19 | 41338988 | G | C | 0.23 | CTC_490E21.12 | 0.99 | -0.01005 |
| rs67210567 | 19 | 41357457 | T | G | 0.03 | CYP2A6 | 0.86 | -0.15082 |
| rs184589612 | 19 | 41412192 | C | T | 0.02 | CTC_490E21.13 | 0.9 | -0.10536 |
| rs12981718 | 19 | 54567858 | A | G | 0.07 | VSTM1 | 0.99 | -0.01005 |
| rs13036436 | 20 | 61988382 | G | A | 0.2 | CHRNA4 | 1.09 | 0.086178 |
| rs61541144 | 20 | 62527305 | A | G | 0.07 | DNAJC5 | 0.91 | -0.09431 |
