# Supplementary material for: Allostatic load, genetic susceptibility, and lung cancer risk: Evidence from a prospective cohort and integrative proteomic analyses
Source: PLOS Digit Health. 2026 Oct 14;5(10):e0001766. doi: 10.1371/journal.pdig.0001766 (PMC13667778; doi:10.1371/journal.pdig.0001766)
Supplement: Supplementary file 2 [file pdig.0001766.s007.docx]

**S2 Table** Distribution of individual biomarkers and AL score category among UK Biobank cohort

| **Characteristic** | **LC free controls** | **LC cases** | ***p value*** |
| --- | --- | --- | --- |
|  | **(n = 333956)** | **(n = 2989)** |  |
| Hemoglobin A1c, mean (SD), mmol/mol | 35.91 (6.51) | 38.14 (6.99) | <0.001 |
| HDL cholesterol, mean (SD), mmol/l | 1.45 (0.38) | 1.39 (0.39) | <0.001 |
| LDL cholesterol, mean (SD), mmol/l | 3.57 (0.87) | 3.43 (0.90) | <0.001 |
| Triglycerides, mean (SD), mmol/l | 1.70 (1.0) | 1.92 (1.05) | <0.001 |
| Systolic blood pressure, mean (SD), mmHg | 139.73 (19.60) | 143.64 (20.30) | <0.001 |
| Diastolic blood pressure, mean (SD), mmHg | 82.22 (10.67) | 81.60 (10.56) | 0.001 |
| Pulse rate, mean (SD), bpm | 69.43 (11.62) | 71.92 (12.46) | <0.001 |
| C-reactive protein, mean (SD), mg/l | 2.55 (4.24) | 4.02 (5.88) | <0.001 |
| Insulin-like growth factor 1, mean (SD), ng/ml | 21.42 (5.62) | 20.00 (5.60) | <0.001 |
| Creatinine, mean (SD), mmol/l | 72.25 (17.37) | 73.71 (28.25) | <0.001 |
| Total cholesterol, mean (SD), mg | 5.71 (1.14) | 5.52 (1.18) | <0.001 |
| Waist-to-height ratio, mean (SD) | 0.87 (0.09) | 0.90 (0.09) | <0.001 |
| AL score, mean (SD) | 3.04 (2.11) | 3.86 (2.06) | <0.001 |
| AL-ternary groups, n (%) |  |  | <0.001 |
| Normal | 37960 (11.4) | 125 (4.2) |  |
| Low | 166516 (49.9) | 1220 (40.8) |  |
| High | 129467 (38.7) | 1644 (55.0) |  |
| P value for trend |  |  | <0.001 |

Abbreviations: AL, allostatic load; SD, standard deviation; HDL, high-lipoprotein cholesterol; LDL, low-density lipoprotein cholesterol.
