# Supplementary material for: Allostatic load, genetic susceptibility, and lung cancer risk: Evidence from a prospective cohort and integrative proteomic analyses
Source: PLOS Digit Health. 2026 Oct 14;5(10):e0001766. doi: 10.1371/journal.pdig.0001766 (PMC13667778; doi:10.1371/journal.pdig.0001766)
Supplement: Supplementary file 3 [file pdig.0001766.s008.docx]

**S3 Table** Association between AL subsystems and the risk of lung cancer

| **AL Subgroup** | **Person-year Incidence (per 1,000 PY, 95% CI) of Lung Cancer Cases** | **HR** | **95% CI** | ***p value*** |
| --- | --- | --- | --- | --- |
| Cardiovascular | 0.236 (0.227,0.244) | 1.054 | (1.014, 1.095) | 0.007 |
| Metabolic | 0.236 (0.227,0.244) | 1.057 | (1.028, 1.086) | <0.001 |
| Inflammatory | 0.236 (0.227,0.244) | 1.301 | (1.235, 1.370) | <0.001 |
| Kidney | 0.236 (0.227,0.244) | 0.926 | (0.846, 1.013) | 0.093 |

The model was adjusted for age, sex, body mass index, smoking status, alcohol intake frequency, International Physical Activity Questionnaire, education level, Townsend deprivation index and family history of lung cancer.

Abbreviations: AL, allostatic load; BMI, body mass index; IPAQ, International Physical Activity Questionnaire; Townsend, Townsend deprivation index; HR, hazard ratio; CI, confidence interval.
