# Supplementary material for: Allostatic load, genetic susceptibility, and lung cancer risk: Evidence from a prospective cohort and integrative proteomic analyses
Source: PLOS Digit Health. 2026 Oct 14;5(10):e0001766. doi: 10.1371/journal.pdig.0001766 (PMC13667778; doi:10.1371/journal.pdig.0001766)
Supplement: Supplementary file 5 [file pdig.0001766.s010.docx]

**S5 Table** Interaction between AL and genetic susceptibility in relation to lung cancer incidence

| **AL Category** | **Term** | **HR** | **95% CI** | ***p value*** |
| --- | --- | --- | --- | --- |
| AL Ternary | Normal AL | Ref |  |  |
|  | Low AL | 1.262 | 0.828–1.923 | 0.279 |
|  | High AL | 1.806 | 1.190–2.740 | 0.005 |
| Low AL | Low AL × Intermediate genetic risk | 0.972 | 0.596–1.584 | 0.908 |
|  | Low AL × High genetic risk | 1.129 | 0.651–1.958 | 0.666 |
| High AL | High AL × Intermediate genetic risk | 0.818 | 0.506–1.324 | 0.414 |
|  | High AL × High genetic risk | 1.003 | 0.582–1.728 | 0.990 |

The model was adjusted for age, sex, body mass index, alcohol intake frequency, International Physical Activity Questionnaire, education level, Townsend deprivation index and family history of lung cancer.

Abbreviations: AL, allostatic load; BMI, body mass index; IPAQ, International Physical Activity Questionnaire; Townsend, Townsend deprivation index; HR, hazard ratio; CI, confidence interval.
