# Supplementary material for: Allostatic load, genetic susceptibility, and lung cancer risk: Evidence from a prospective cohort and integrative proteomic analyses
Source: PLOS Digit Health. 2026 Oct 14;5(10):e0001766. doi: 10.1371/journal.pdig.0001766 (PMC13667778; doi:10.1371/journal.pdig.0001766)
Supplement: Supplementary file 6 [file pdig.0001766.s011.docx]

**S6 Table** RERI, AP and SI for additive interaction between AL and genetic categories

The model was adjusted for age, sex, body mass index, smoking status, alcohol intake frequency, International Physical Activity Questionnaire, education level, Townsend deprivation index and family history of lung cancer.

Abbreviations: AL, allostatic load; RERI: Relative excess risk due to interaction; AP: Attributable proportion

due to interaction; SI: Synergy index; PRS: Polygenic risk score; CI, confidence interval.

| **Strata** | **RERI (95% CI)** | **AP (95% CI)** | **SI (95% CI)** |
| --- | --- | --- | --- |
| **Medium genetic risk** |  |  |  |
| Low AL | 0.050 (-0.494, 0.593) | 0.029 (-0.300, 0.359) | 1.078 (0.437, 2.655) |
| High AL | -0.148 (-0.759, 0.462) | -0.073 (-0.353, 0.207) | 0.875 (0.557, 1.374) |
| **High genetic risk** |  |  |  |
| Low AL | 0.293 (-0.293, 0.879) | 0.157 (-0.187, 0.502) | 1.515 (0.422, 5.439) |
| High AL | 0.256 (0.352, 0.863) | 0.108 (-0.169, 0.385) | 1.230 (0.656, 2.306) |
