# Supplementary material for: Allostatic load, genetic susceptibility, and lung cancer risk: Evidence from a prospective cohort and integrative proteomic analyses
Source: PLOS Digit Health. 2026 Oct 14;5(10):e0001766. doi: 10.1371/journal.pdig.0001766 (PMC13667778; doi:10.1371/journal.pdig.0001766)
Supplement: Supplementary file 8 [file pdig.0001766.s013.docx]

**S8 Table** Lung cancer risk according to NLST eligibility and AL category

| **AL Category** | **NLST eligibility** | **Person-year Incidence (per 1,000 PY, 95% CI) of LC Cases** | | **HR** | **95% CI** | ***p*-value** |
| --- | --- | --- | --- | --- | --- | --- |
| AL-Continuous | Not eligible | 0.570 (0.546, 0.595) | 1.055 | | (1.031, 1.080) | <0.001 |
|  | Eligible | 6.294 (5.972, 6.630) | 1.039 | | (1.011, 1.067) | 0.006 |
| AL-Ternary |  |  |  | |  |  |
| Normal | Not eligible | 0.313 (0.263, 0.371) | Ref | |  |  |
| Low | Not eligible | 0.510 (0.478, 0.544) | 1.109 | | (0.924, 1.331) | 0.268 |
| High | Not eligible | 0.735 (0.690, 0.782) | 1.330 | | (1.102, 1.604) | 0.003 |
| Normal | Eligible | 3.822 (2.540, 5.524) | 2.020 | | (1.339, 3.046) | <0.001 |
| Low | Eligible | 5.937 (5.425, 6.484) | 2.963 | | (2.424, 3.621) | <0.001 |
| High | Eligible | 6.647 (6.221, 7.095) | 3.414 | | (2.804, 4.158) | <0.001 |

The model was adjusted for age, sex, body mass index, smoking status, alcohol intake frequency, indicates International Physical Activity Questionnaire, education level, Townsend deprivation index and family history of lung cancer.

Abbreviations: AL, allostatic load; PY, person-year; HR, hazard ratio; CI, confidence interval; NLST, National Lung Screening Trial.
