# Supplementary material for: Allostatic load, genetic susceptibility, and lung cancer risk: Evidence from a prospective cohort and integrative proteomic analyses
Source: PLOS Digit Health. 2026 Oct 14;5(10):e0001766. doi: 10.1371/journal.pdig.0001766 (PMC13667778; doi:10.1371/journal.pdig.0001766)
Supplement: Supplementary file 9 [file pdig.0001766.s014.docx]

**S9 Table** Incremental discrimination after adding AL to LDCT screening eligibility models

| **Model** | **C-index base (95% CI)** | **C-index + AL (95% CI)** | | **ΔC-index** | ***p for LRT*** |
| --- | --- | --- | --- | --- | --- |
| USPSTF 2021 | 0.723 (0.715, 0.731) | 0.755 (0.746, 0.764) | 0.032 | | <0.001 |
| NLST | 0.677 (0.669, 0.686) | 0.721 (0.712, 0.731) | 0.044 | | <0.001 |
| Clinical model | 0.836 (0.830, 0.841) | 0.839 (0.833, 0.846) | 0.004 | | <0.001 |

**Note:** C-index values were calculated from Cox proportional hazards models. The base models included USPSTF 2021 eligibility, NLST eligibility, or clinical covariates, respectively. The +AL models additionally included continuous AL score. The clinical model included age, sex, body mass index, smoking status, alcohol intake frequency, International Physical Activity Questionnaire, education level, Townsend deprivation index, and family history of lung cancer.
**Abbreviations:** AL, allostatic load; LDCT, low-dose computed tomography; USPSTF, United States Preventive Services Task Force; NLST, National Lung Screening Trial; CI, confidence interval; LRT, likelihood ratio test.
