# Supplementary material for: Allostatic load, genetic susceptibility, and lung cancer risk: Evidence from a prospective cohort and integrative proteomic analyses
Source: PLOS Digit Health. 2026 Oct 14;5(10):e0001766. doi: 10.1371/journal.pdig.0001766 (PMC13667778; doi:10.1371/journal.pdig.0001766)
Supplement: Supplementary file 10 [file pdig.0001766.s015.docx]

**S10 Table** Subgroups analyses of the associations between AL (three-categories, normal as reference) and the risk of lung cancer

| **Subgroup** | **Level** | **Low AL** | | | **High AL** | | |  |
| --- | --- | --- | --- | --- | --- | --- | --- | --- |
|  |  | **Incidence**  **(per 1,000 PY, 95% CI)** | **HR**  **(95% CI)** | **p value** | **Incidence**  **(per 1,000 PY, 95% CI)** | **HR**  **(95% CI)** | ***p*-value** | ***p for***  ***interaction*** |
| Gender | Male | 0.541  (0.495, 0.591) | 1.342  (0.921, 1.954) | 0.125 | 1.015  (0.955, 1.078) | 1.905  (1.309, 2.772) | <0.001 | 0.020 |
|  | Female | 0.538  (0.500, 0.579) | 1.321  (1.064, 1.641) | 0.012 | 0.848  (0.780, 0.920) | 1.489  (1.186, 1.871) | <0.001 |  |
| Age (years) | <60 | 0.299  (0.271, 0.328) | 1.646  (1.279, 2.119) | <0.001 | 0.542  (0.496, 0.591) | 2.707  (2.076, 3.528) | <0.001 | 0.342 |
|  | ≥60 | 0.974  (0.907, 1.045) | 1.427  (1.080, 1.887) | 0.012 | 1.430  (1.348, 1.516) | 1.763  (1.331, 2.336) | <0.001 |  |
| BMI | <28 | 0.551  (0.515, 0.588) | 1.285  (1.048, 1.575) | 0.016 | 1.015  (0.943, 1.091) | 1.526  (1.237, 1.883) | <0.001 | 0.136 |
|  | ≥28 | 0.512  (0.459, 0.570) | 1.110  (0.689, 1.786) | 0.669 | 0.901  (0.844, 0.962) | 1.441  (0.899, 2.309) | 0.129 |  |
| Ethnicity | White | 0.543  (0.513, 0.575) | 1.276  (1.058, 1.539) | 0.011 | 0.956  (0.910, 1.003) | 1.616  (1.334, 1.956) | <0.001 | 0.657 |
|  | Non-White | 0.317  (0.152, 0.583) | 1.731  (0.211, 14.190) | 0.609 | 0.430  (0.214, 0.769) | 2.249  (0.253, 20.026) | 0.468 |  |
| Smoking Status | Never | 0.142  (0.122, 0.164) | 0.752  (0.533, 1.062) | 0.105 | 0.187  (0.159, 0.218) | 0.882  (0.608, 1.279) | 0.506 | <0.001 |
|  | Previous | 0.743  (0.683, 0.807) | 1.327  (0.998, 1.765) | 0.052 | 1.194  (1.112, 1.281) | 1.673  (1.251, 2.237) | <0.001 |  |
|  | Current | 2.185  (1.990, 2.394) | 1.861  (1.283, 2.700) | 0.001 | 3.237  (2.999, 3.489) | 2.448  (1.681, 3.564) | <0.001 |  |
| Alcohol Intake | Low & Moderate | 0.555  (0.510, 0.602) | 1.505  (1.132, 2.000) | 0.005 | 0.951  (0.883, 1.023) | 1.985  (1.484, 2.655) | <0.001 | 0.164 |
|  | Heavy | 0.528  (0.488, 0.570) | 1.141  (0.892, 1.461) | 0.294 | 0.943  (0.883, 1.006) | 1.414  (1.099, 1.819) | 0.007 |  |
| IPAQ Group | Low & Moderate | 0.533  (0.490, 0.579) | 1.497  (1.115, 2.011) | 0.007 | 0.930  (0.867, 0.996) | 1.881  (1.393, 2.540) | <0.001 | 0.294 |
|  | Heavy | 0.497  (0.449, 0.548) | 1.046  (0.780, 1.404) | 0.764 | 0.884  (0.804, 0.970) | 1.321  (0.975, 1.790) | 0.073 |  |
| College/University Degree | Yes | 0.277  (0.242, 0.317) | 1.141  (0.792, 1.643) | 0.479 | 0.451  (0.392, 0.517) | 1.427  (0.970, 2.099) | 0.071 | 0.622 |
|  | No | 0.671  (0.629, 0.714) | 1.329  (1.067, 1.654) | 0.011 | 1.126  (1.068, 1.186) | 1.701  (1.362, 2.125) | <0.001 |  |
| Townsend Index | ≤ median | 0.397  (0.362, 0.435) | 1.122  (0.843, 1.493) | 0.429 | 0.678  (0.624, 0.736) | 1.430  (1.067, 1.916) | 0.017 | 0.553 |
|  | > median | 0.691  (0.642, 0.742) | 1.453  (1.136, 1.858) | 0.003 | 1.205  (1.134, 1.279) | 1.886  (1.468, 2.424) | <0.001 |  |
| Family History of lung Cancer | Yes | 1.014  (0.901, 1.138) | 1.784  (1.141, 2.791) | 0.011 | 1.511  (1.360, 1.675) | 2.046  (1.297, 3.229) | 0.002 | 0.186 |
|  | No | 0.471  (0.441, 0.502) | 1.214  (0.988, 1.490) | 0.065 | 0.858  (0.812, 0.906) | 1.619  (1.313, 1.997) | <0.001 |  |

The model was adjusted for age, sex, body mass index, smoking status, alcohol intake frequency, IPAQ, education level, Townsend deprivation index and family history of lung cancer, without adjustment for the corresponding items.

Abbreviations: AL, allostatic load; BMI, body mass index; IPAQ, International Physical Activity Questionnaire; Townsend, Townsend deprivation index; HR, hazard ratio; CI, confidence interval.
