# Supplementary material for: Allostatic load, genetic susceptibility, and lung cancer risk: Evidence from a prospective cohort and integrative proteomic analyses
Source: PLOS Digit Health. 2026 Oct 14;5(10):e0001766. doi: 10.1371/journal.pdig.0001766 (PMC13667778; doi:10.1371/journal.pdig.0001766)
Supplement: Supplementary file 11 [file pdig.0001766.s016.docx]

**S11 Table** Interaction between AL and smoking status in relation to lung cancer incidence

| **AL Category** | **Term** | **HR** | **95% CI** | ***p value*** |
| --- | --- | --- | --- | --- |
| AL-Continuous | AL among never smokers | 0.987 | (0.938, 1.039) | 0.614 |
|  | AL **×** Former smokers | 1.111 | (1.051, 1.175) | <0.001 |
|  | AL **×** Current smokers | 1.087 | (1.027, 1.150) | 0.004 |
| AL-Ternary | Normal among never smokers | Ref |  |  |
|  | Low among never smokers | 0.679 | (0.485, 0.951) | 0.024 |
|  | High among never smokers | 0.748 | (0.531, 1.055) | 0.098 |
|  | Low AL × Former smokers | 2.107 | (1.360, 3.266) | <0.001 |
|  | High AL × Former smokers | 2.587 | (1.668, 4.014) | <0.001 |
|  | Low AL × Current smokers | 2.658 | (1.614, 4.378) | <0.001 |
|  | High AL × Current smokers | 2.982 | (1.810, 4.913) | <0.001 |

The model was adjusted for age, sex, body mass index, alcohol intake frequency, International Physical Activity Questionnaire, education level, Townsend deprivation index and family history of lung cancer.

Abbreviations: AL, allostatic load; BMI, body mass index; IPAQ, International Physical Activity Questionnaire; Townsend, Townsend deprivation index; HR, hazard ratio; CI, confidence interval.
