# Supplementary material for: Allostatic load, genetic susceptibility, and lung cancer risk: Evidence from a prospective cohort and integrative proteomic analyses
Source: PLOS Digit Health. 2026 Oct 14;5(10):e0001766. doi: 10.1371/journal.pdig.0001766 (PMC13667778; doi:10.1371/journal.pdig.0001766)
Supplement: Supplementary file 12 [file pdig.0001766.s017.docx]

**S12 Table** Sensitivity analysis for association between AL and lung cancer incidence additionally adjusted for smoking related covariates

| **AL Category** | **Person-year Incidence (per 1,000 PY, 95% CI) of Lung Cancer Cases** | **HR** | **95% CI** | ***p value*** |
| --- | --- | --- | --- | --- |
| AL-Continuous | 0.654 (0.631, 0.678) | 1.039 | (1.018, 1.061) | <0.001 |
| AL-Ternary |  |  |  |  |
| Normal | 0.241 (0.201, 0.287) | Ref |  |  |
| Low | 0.533 (0.504, 0.564) | 1.186 | (0.970, 1.451) | 0.097 |
| High | 0.937 (0.892, 0.984) | 1.370 | (1.114, 1.684) | 0.003 |

The model was adjusted for age, sex, body mass index, smoking status, alcohol intake frequency, International Physical Activity Questionnaire, education level, Townsend deprivation index, family history of lung cancer, smoking duration (years), number of cigarettes smoked per day, and years since quitting smoking.

Abbreviations: AL, allostatic load; PY, person-year; HR, hazard ratio; CI, confidence interval.
