# Supplementary material for: Allostatic load, genetic susceptibility, and lung cancer risk: Evidence from a prospective cohort and integrative proteomic analyses
Source: PLOS Digit Health. 2026 Oct 14;5(10):e0001766. doi: 10.1371/journal.pdig.0001766 (PMC13667778; doi:10.1371/journal.pdig.0001766)
Supplement: Supplementary file 13 [file pdig.0001766.s018.docx]

**S13 Table** Sensitivity analysis for association between AL and lung cancer subtypes additionally adjusted for smoking related covariates

| **Lung Cancer Subtype** | **Person-year Incidence (per 1,000 PY, 95% CI) of Cases** | **Hazard Ratio** | **95% CI** | ***p value*** |
| --- | --- | --- | --- | --- |
| LUAD |  |  |  |  |
| AL-Continuous | 0.283 (0.268, 0.299) | 1.023 | (0.991, 1.057) | 0.161 |
| AL-Ternary |  |  |  |  |
| Normal | 0.140 (0.110, 0.177) | Ref |  |  |
| Low | 0.243 (0.223, 0.264) | 0.980 | (0.754, 1.275) | 0.881 |
| High | 0.378 (0.350, 0.408) | 1.119 | (0.852, 1.470) | 0.034 |
| LUSC |  |  |  |  |
| AL-Continuous | 0.175 (0.163, 0.188) | 1.041 | (1.001, 1.084) | 0.049 |
| AL-Ternary |  |  |  |  |
| Normal | 0.040 (0.025, 0.062) | Ref |  |  |
| Low | 0.131 (0.117, 0.147) | 1.734 | (1.061, 2.591) | 0.035 |
| High | 0.273 (0.249, 0.299) | 2.073 | (1.239, 3.466) | 0.005 |
| SCLC |  |  |  |  |
| AL-Continuous | 0.055 (0.049, 0.063) | 1.081 | (1.009, 1.158) | 0.027 |
| AL-Ternary |  |  |  |  |
| Normal | 0.010 (0.003, 0.022) | Ref |  |  |
| Low | 0.042 (0.034, 0.051) | 1.932 | (0.963, 5.884) | 0.156 |
| High | 0.088 (0.074, 0.103) | 2.471 | (1.056, 5.782) | 0.037 |

The model was adjusted for age, sex, body mass index, smoking status, alcohol intake frequency, International Physical Activity Questionnaire, education level, Townsend deprivation index, family history of lung cancer, smoking duration (years), number of cigarettes smoked per day, and years since quitting smoking.

Abbreviations: AL, allostatic load; PY, person-year; HR, hazard ratio; CI, confidence interval; LUAD, Lung Adenocarcinoma; LUSC, Lung Squamous Cell Carcinoma; SCLC, Small Cell Lung Cancer.
