# Supplementary material for: Allostatic load, genetic susceptibility, and lung cancer risk: Evidence from a prospective cohort and integrative proteomic analyses
Source: PLOS Digit Health. 2026 Oct 14;5(10):e0001766. doi: 10.1371/journal.pdig.0001766 (PMC13667778; doi:10.1371/journal.pdig.0001766)
Supplement: Supplementary file 14 [file pdig.0001766.s019.docx]

**S14 Table** Sensitivity analysis for association between AL and lung cancer incidence excluding the population diagnosed with chronic obstructive pulmonary disease at enrollment

| **AL Category** | **Person-year Incidence (per 1,000 PY, 95% CI) of Lung Cancer Cases** | **HR** | **95% CI** | ***p value*** |
| --- | --- | --- | --- | --- |
| AL-Continuous | 0.636 (0.613, 0.660) | 1.074 | (1.053, 1.095) | <0.001 |
| AL-Ternary |  |  |  |  |
| Normal | 0.233 (0.193, 0.278) | Ref |  |  |
| Low | 0.521 (0.492, 0.552) | 1.293 | (1.069, 1.562) | 0.008 |
| High | 0.908 (0.864, 0.954) | 1.640 | (1.351, 1.990) | <0.001 |

The model was adjusted for age, sex, body mass index, smoking status, alcohol intake frequency, International Physical Activity Questionnaire, education level, Townsend deprivation index and family history of lung cancer.

Abbreviations: AL, allostatic load; PY, person-year; HR, hazard ratio; CI, confidence interval.
