# Supplementary material for: Allostatic load, genetic susceptibility, and lung cancer risk: Evidence from a prospective cohort and integrative proteomic analyses
Source: PLOS Digit Health. 2026 Oct 14;5(10):e0001766. doi: 10.1371/journal.pdig.0001766 (PMC13667778; doi:10.1371/journal.pdig.0001766)
Supplement: Supplementary file 17 [file pdig.0001766.s022.docx]

**S17 Table** Sensitivity analysis for association between AL and lung cancer Subtype Incidence using Firth’s Cox Regression

| **Lung Cancer Subtype** | **Person-year Incidence (per 1,000 PY, 95% CI) of Cases** | **Hazard Ratio** | **95% CI** | ***p value*** |
| --- | --- | --- | --- | --- |
| LUAD |  |  |  |  |
| AL-Continuous | 0.283 (0.268, 0.299) | 1.048 | (1.018, 1.080) | 0.002 |
| AL-Ternary |  |  |  |  |
| Normal | 0.140 (0.110, 0.177) | Ref |  |  |
| Low | 0.243 (0.223, 0.264) | 1.058 | (0.832, 1.366) | 0.652 |
| High | 0.378 (0.350, 0.408) | 1.294 | (1.008, 1.685) | 0.043 |
| LUSC |  |  |  |  |
| AL-Continuous | 0.175 (0.163, 0.188) | 1.088 | (1.047, 1.130) | <0.001 |
| AL-Ternary |  |  |  |  |
| Normal | 0.040 (0.025, 0.062) | Ref |  |  |
| Low | 0.131 (0.117, 0.147) | 1.935 | (1.227, 3.266) | 0.003 |
| High | 0.273 (0.249, 0.299) | 2.608 | (1.646, 4.419) | <0.001 |
| SCLC |  |  |  |  |
| AL-Continuous | 0.055 (0.049, 0.063) | 1.116 | (1.046, 1.189) | <0.001 |
| AL-Ternary |  |  |  |  |
| Normal | 0.010 (0.003, 0.022) | Ref |  |  |
| Low | 0.042 (0.034, 0.051) | 2.176 | (1.005, 5.871) | 0.048 |
| High | 0.088 (0.074, 0.103) | 2.987 | (1.368, 8.103) | 0.004 |
| LCC |  |  |  |  |
| AL-Continuous | 0.007 (0.004, 0.010) | 1.077 | (0.884, 1.301) | NA |
| AL-Ternary |  |  |  |  |
| Normal | 0.002 (0.000, 0.011) | Ref |  |  |
| Low | 0.006 (0.003, 0.010) | 1.306 | (0.311, 12.095) | NA |
| High | 0.009 (0.005, 0.015) | 1.258 | (0.274, 12.128) | NA |

The model was adjusted for age, sex, body mass index, smoking status, alcohol intake frequency, International Physical Activity Questionnaire, education level, Townsend deprivation index and family history of lung cancer, without adjustment for the corresponding items.

Abbreviations: AL, allostatic load; LUAD, Lung Adenocarcinoma; LUSC, Lung Squamous Cell Carcinoma; SCLC, Small Cell Lung Cancer; LCC, Large Cell Carcinoma; PY, person-year; HR, hazard ratio; CI, confidence interval.
