# Supplementary material for: Allostatic load, genetic susceptibility, and lung cancer risk: Evidence from a prospective cohort and integrative proteomic analyses
Source: PLOS Digit Health. 2026 Oct 14;5(10):e0001766. doi: 10.1371/journal.pdig.0001766 (PMC13667778; doi:10.1371/journal.pdig.0001766)
Supplement: Supplementary file 18 [file pdig.0001766.s023.docx]

**S18 Table** Association of AL with risk of lung cancer mediated by individual protein

| **Outcome** | **Exposure** | **Protein** | **Proportion (%)** | **95% CI (%)** | ***p value*** |
| --- | --- | --- | --- | --- | --- |
| Lung Cancer | AL | HGF | 27.90 | (13.5, 72.5) | <0.001 |
| Lung Cancer | AL | RARRES2 | 22.74 | (5.2, 60.6) | <0.001 |
| Lung Cancer | AL | GDF15 | 22.34 | (13.4, 60.2) | <0.001 |
| Lung Cancer | AL | APCS | 19.85 | (6.2, 56.6) | <0.001 |
| Lung Cancer | AL | FGF21 | 17.25 | (6.1, 51.3) | <0.001 |
| Lung Cancer | AL | CFH | 16.76 | (4.3, 41.9) | 0.020 |
| Lung Cancer | AL | PTPRB | 16.52 | (6.4, 50.1) | <0.001 |
| Lung Cancer | AL | LECT2 | 16.41 | (7.5, 52.3) | <0.001 |
| Lung Cancer | AL | MSR1 | 13.58 | (4.7, 32.9) | 0.020 |
| Lung Cancer | AL | HAVCR1 | 13.56 | (5.2, 37.7) | <0.001 |
| Lung Cancer | AL | ADM | 13.47 | (4.2, 35.9) | <0.001 |
| Lung Cancer | AL | DSG2 | 11.88 | (2.6, 43.2) | <0.001 |
| Lung Cancer | AL | IL6 | 11.67 | (4.6, 27.4) | 0.020 |
| Lung Cancer | AL | MXRA8 | 10.81 | (1.0, 40.4) | 0.040 |
| Lung Cancer | AL | PLAT | 10.65 | (0.4, 31.0) | 0.040 |
| Lung Cancer | AL | LRIG1 | 7.10 | (3.1, 17.3) | <0.001 |
| Lung Cancer | AL | KITLG | 6.94 | (1.0, 23.2) | 0.020 |
| Lung Cancer | AL | SPINK2 | 6.26 | (0.1, 19.2) | 0.040 |
| Lung Cancer | AL | VWC2 | 5.91 | (1.39, 17.24) | <0.001 |
| Lung Cancer | AL | BCAN | 4.53 | (1.9, 12.43) | <0.001 |
| Lung Cancer | AL | APOA1 | 3.14 | (0.56, 8.53) | 0.020 |
| Lung Cancer | AL | REN | 2.94 | (0.93, 8.95) | <0.001 |
| Lung Cancer | AL | IL15 | 2.06 | (0.28, 6.22) | 0.040 |
| Lung Cancer | AL | NTRK3 | 1.60 | (0.12, 5.89) | 0.040 |
| Lung Cancer | AL | NCAN | 1.12 | (0.17, 3.15) | <0.001 |
| Lung Cancer | AL | AGER | -1.24 | (-3.08, -0.07) | 0.040 |
| Lung Cancer | AL | DCBLD2 | -1.76 | (-4.68, -0.48) | <0.001 |
| Lung Cancer | AL | IGFBP3 | -2.99 | (-8.44, -0.83) | <0.001 |
| Lung Cancer | AL | TSHB | -3.40 | (-9.12, -0.52) | 0.020 |
| Lung Cancer | AL | ACAN | -3.96 | (-12.28, -1.88) | <0.001 |
| Lung Cancer | AL | COL4A1 | -4.09 | (-10.11, -0.44) | 0.040 |
| Lung Cancer | AL | FGFBP1 | -5.10 | (-12.83, -1.96) | <0.001 |
| Lung Cancer | AL | MSTN | -6.36 | (-16.7, -2.86) | <0.001 |
| Lung Cancer | AL | NPY | -14.96 | (-42.99, -3.26) | 0.020 |

The model was adjusted for age, sex, body mass index, smoking status, alcohol intake frequency, International Physical Activity Questionnaire, education level, Townsend deprivation index and family history of lung cancer.

Abbreviations: AL, allostatic load; CI, confidence interval.
