# Supplementary material for: Allostatic load, genetic susceptibility, and lung cancer risk: Evidence from a prospective cohort and integrative proteomic analyses
Source: PLOS Digit Health. 2026 Oct 14;5(10):e0001766. doi: 10.1371/journal.pdig.0001766 (PMC13667778; doi:10.1371/journal.pdig.0001766)
Supplement: Supplementary file 19 [file pdig.0001766.s024.docx]

**S19 Table** Subtype-specific mediation analysis of protein mediators in NSCLC.

| **Outcome** | **Exposure** | **Protein** | **Proportion (%)** | **95% CI** | ***p_value*** |
| --- | --- | --- | --- | --- | --- |
| LUAD | Protein | DSG2 | 42.3 | (0.006, 0.036) | 0.0040 |
| LUAD | Protein | HGF | 81.7 | (0.032, 0.076) | <0.001 |
| LUAD | Protein | ACAN | -0.9 | (-0.012, -0.003) | <0.001 |
| LUAD | Protein | IL6 | 44.5 | (0.012, 0.032) | <0.001 |
| LUAD | Protein | MSTN | -4.4 | (-0.021, -0.005) | <0.001 |
| LUAD | Protein | GDF15 | 84.6 | (0.026, 0.045) | <0.001 |
| LUAD | Protein | LECT2 | 142.4 | (0.01, 0.041) | <0.001 |
| LUAD | Protein | ADM | 42.3 | (0.013, 0.046) | <0.001 |
| LUAD | Protein | FGF21 | 64.2 | (0.014, 0.057) | <0.001 |
| LUAD | Protein | MXRA8 | 0.5 | (0.009, 0.043) | <0.001 |
| LUAD | Protein | BCAN | 48 | (0.009, 0.018) | <0.001 |
| LUAD | Protein | NTRK3 | 4.6 | (0.003, 0.009) | <0.001 |
| LUAD | Protein | RARRES2 | 119.3 | (0.015, 0.056) | 0.0040 |
| LUAD | Protein | NCAN | 1 | (0.001, 0.007) | 0.0040 |
| LUAD | Protein | HAVCR1 | 171.8 | (0.012, 0.048) | <0.001 |
| SCC | Protein | ESM1 | 32.9 | (0.01, 0.036) | <0.001 |
| SCC | Protein | APOC1 | -43.7 | (-0.021, -0.004) | <0.001 |
| SCC | Protein | HGF | 133.1 | (0.04, 0.096) | <0.001 |
| SCC | Protein | ACAN | -11.6 | (-0.016, -0.004) | 0.0040 |
| SCC | Protein | IL6 | 12.4 | (0.011, 0.034) | <0.001 |
| SCC | Protein | TSHB | -33.5 | (-0.015, -0.004) | <0.001 |
| SCC | Protein | MSTN | 47.9 | (-0.025, -0.006) | <0.001 |
| SCC | Protein | GDF15 | 96.7 | (0.036, 0.055) | <0.001 |
| SCC | Protein | LRIG1 | 32.2 | (0.008, 0.027) | <0.001 |
| SCC | Protein | IGFBP3 | -70.3 | (-0.01, -0.002) | 0.0040 |
| SCC | Protein | ADM | 37.7 | (0.016, 0.064) | 0.0040 |
| SCC | Protein | REN | 2 | (0.002, 0.008) | 0.0040 |
| SCC | Protein | LDLR | -134.3 | (-0.121, -0.023) | 0.0080 |
| SCC | Protein | BCAN | 18.3 | (0.004, 0.017) | <0.001 |
| SCC | Protein | NTRK3 | 4 | (0.002, 0.01) | <0.001 |
| SCC | Protein | IL15 | 8.3 | (0.006, 0.014) | <0.001 |
| SCC | Protein | PTPRB | 356 | (0.023, 0.074) | <0.001 |
| SCC | Protein | APCS | 100.2 | (0.016, 0.089) | 0.0040 |
| SCC | Protein | NCAN | 7.5 | (0.001, 0.007) | <0.001 |
| SCLC | Protein | GDF15 | 61 | (0.032, 0.057) | <0.001 |
| SCLC | Protein | LECT2 | 30.3 | (0.023, 0.063) | <0.001 |
| SCLC | Protein | KITLG | 30.8 | (0.019, 0.051) | <0.001 |
| SCLC | Protein | BCAN | 10.4 | (0.003, 0.019) | <0.001 |
| SCLC | Protein | CDH2 | 75.5 | (-0.065, -0.014) | <0.001 |
| Data are presented as proportion mediated (PM) with 95% confidence interval (CI) and p-value.  Abbreviations: NSCLC, non-small cell lung cancer; LUAD, lung adenocarcinoma; SCC, squamous cell carcinoma; SCLC, small cell lung cancer; PM, proportion mediated; CI, confidence interval. | | | | | |
