# Supplementary material for: Allostatic load, genetic susceptibility, and lung cancer risk: Evidence from a prospective cohort and integrative proteomic analyses
Source: PLOS Digit Health. 2026 Oct 14;5(10):e0001766. doi: 10.1371/journal.pdig.0001766 (PMC13667778; doi:10.1371/journal.pdig.0001766)
Supplement: Supplementary file 20 [file pdig.0001766.s025.docx]

# Response to Reviewers

Dear Editor,

We thank the editor and all three reviewers for the constructive and insightful comments, which have greatly helped us improve the rigor, clarity, and scope of the manuscript. We have carefully addressed each comment point-by-point, revised the manuscript accordingly, and strengthened causal language restraint, smoking-related sensitivity analyses, methodological justifications, novelty clarification, and digital health relevance as requested. Below we provide detailed responses to each reviewer’s comments.

## Responses to Reviewer #1

### Introduction

**Comment 1:** Tone down the language stating that plasma proteomics “elucidate” causal pathways; frame analyses as exploratory and hypothesis-generating rather than mechanistic proof.

**Response:** We appreciate this important comment. Modified as requested. We have revised the manuscript to explicitly frame plasma proteomic analyses as exploratory and hypothesis-generating, and toned down causal language throughout. We no longer state that plasma proteomics “elucidate” causal pathways. Instead, we clarify that these analyses identify candidate biological correlates and potential mediators for hypothesis generation.

**Comment 2**: Acknowledge AL as a composite biomarker overlapping with behavioral risk profiles, not purely a modifiable determinant.

**Response**:We agree with the reviewer that AL should be more carefully framed as a composite biomarker rather than simply as a modifiable determinant. We have revised the Introduction and Discussion sections to explicitly recognize AL as a composite biomarker of multisystem physiological dysregulation that overlaps with behavioral risk profiles, rather than presenting it purely as a modifiable determinant.

**Comment 3:** Recognize AL may function as a correlate of cumulative disadvantage (smoking, socioeconomic factors) rather than a distinct causal driver.

**Response:** We agree with the reviewer that the association between AL and lung cancer risk should be interpreted cautiously, as AL may partly capture cumulative behavioral, socioeconomic, and environmental disadvantage rather than represent a distinct causal driver. We have updated the manuscript to explicitly acknowledge that AL may reflect cumulative disadvantage, rather than acting as a distinct causal driver of lung cancer. The modifications are as follows:

“As an integrative measure of physiological dysregulation, AL may also reflect long-term exposure to behavioral, socioeconomic, and environmental disadvantages.”

### Methods

**Comment 4:** Address exclusion of 126,829 individuals due to missing AL biomarkers and potential selection bias; compare baseline characteristics between included and excluded participants. This is a substantial proportion of the original cohort and naturally raises concerns about selection bias.

**Response:** We appreciate the reviewer’s insightful comment regarding potential selection bias. To address this concern, we compared baseline characteristics between participants included in the analysis and those excluded. Standardized mean differences (SMDs) were used to evaluate group differences, with values <0.1 indicating negligible imbalance. Overall, the characteristics were comparable between the two groups, suggesting that selection bias is unlikely to have materially affected our findings. The relevant table has been provided at the end of this response letter.

·Table 1. Baseline characteristics stratified by group

**Comment 5:** Provide detailed rationale for AL score construction; justify defining AL = 0 as “normal” and splitting AL > 0 by median; test robustness under alternative AL definitions.

**Response:** We appreciate the reviewer’s insightful comment. We agree that the categorization of AL should be biologically interpretable and methodologically justified. In our study, AL = 0 was defined as a biologically meaningful reference group, representing individuals without any detectable physiological dysregulation across the included systems. Unlike approaches that dichotomize AL across the entire population, this strategy avoids treating individuals with no detectable dysregulation as equivalent to those with mild but existing physiological burden. Given the absence of universally accepted cutoffs for AL, we used the median among participants with AL > 0 to further stratify participants with AL > 0 into lower and higher burden groups. Importantly, we conducted additional sensitivity analyses using continuous AL, which yielded consistent results, supporting the robustness of our findings.

**Comment 6:** Temporality and Technical Effects: How were batch effects and multiple testing controlled for in the proteomic subset?

**Response:** We have revised the Methods section to clarify how technical effects were addressed in the proteomic analyses. The UKB-PPP proteomic data were generated using the Olink Explore 3072 platform and processed through a standardized quality-control and normalization pipeline. Previous UKB-PPP quality-control analyses reported no global batch or plate effects after processing, with less than 10% variability attributable to batch for 100% of proteins and to plate for 99.5% of proteins [22]. In addition, we excluded proteins with high missingness, applied imputation only to proteins with missingness below the predefined threshold, and controlled multiple testing using false discovery rate correction in downstream enrichment and mediation analyses.

**Reference**

[22] Sun BB, Chiou J, Traylor M, Benner C, Hsu YH, Richardson TG, et al. Plasma proteomic associations with genetics and health in the UK Biobank. Nature. 2023;622(7982):329-38.

**Comment 7:** Mediation Assumptions: the assumptions required for formal mediation are difficult to satisfy in this observational context.

**Response:** We thank the reviewer for raising this important methodological point. We agree that the assumptions required for formal causal mediation analysis are difficult to fully satisfy in an observational study, particularly given the possibility of residual confounding and temporal ambiguity. We have therefore revised the manuscript to clarify that the mediation analyses should be interpreted as exploratory association-based analyses rather than evidence of causal mediation. The following statement has been added to the Methods/Discussion section:

“Given the observational design, potential residual confounding, and the difficulty of verifying the assumptions required for formal causal mediation analysis, these analyses were considered exploratory and should not be interpreted as evidence of causal mediation.”

**Comment 8:** Machine Learning Rigor: It would be helpful to clarify if feature selection and SHAP ranking were fully nested within cross-validation to prevent data leakage, and whether the identified proteins were validated in an independent subset.

**Response:** We thank the reviewer for this important methodological concern. In our revised manuscript, we have clarified that the feature selection step (using a machine learning framework) was not nested within cross‑validation, as its purpose was to identify AL‑associated proteins in an exploratory manner rather than to build a predictive model for generalization. To prevent overfitting and to evaluate robustness, we performed bootstrap stability analysis with 100 iterations. Selection frequency was calculated for each of the 111 proteins across the bootstrap iterations, and all 111 proteins were repeatedly selected in more than 80 of 100 iterations, indicating high stability. Regarding independent validation, we acknowledge the absence of an external validation set and have added a limitation statement in the Discussion, noting that the identified protein panel requires confirmation in independent proteomic cohorts.

### Results

**Comment 9:** Avoid overinterpreting omics results; label proteins (HGF, RARRES2, GDF15) as markers of inflammation, smoking burden, or preclinical disease, not proven mechanistic transmitters.

**Response:** We fully agree with the reviewer that the identified proteins should not be interpreted as proven mechanistic transmitters. We have revised the Discussion section to avoid causal or mechanistic overstatement and now describe HGF, RARRES2, and GDF15 as exploratory protein markers that may reflect inflammation, smoking-related biological burden, or preclinical disease processes. The specific wording has been revised to: “Examining these AL-associated proteins may help characterize biological processes correlated with AL and lung cancer risk, but these findings should be interpreted as exploratory and do not establish mechanistic transmission.”

**Discussion and Conclusion**

**Comment 10:** Refer to AL as a risk marker, not a validated prediction tool, without demonstrating incremental predictive value over existing factors.

**Response:** We agree with the reviewer that AL should not be presented as a validated prediction tool. In response to this concern, we incorporated established LDCT screening eligibility criteria, including the USPSTF 2021 and NLST criteria, and evaluated whether adding AL improved risk discrimination beyond these rule-based screening frameworks. We also assessed the incremental value of AL beyond a multivariable clinical model including demographic, clinical, and smoking-related covariates. Although these analyses suggested that AL may improve risk stratification, particularly when added to rule-based screening eligibility criteria, we recognize that this does not constitute formal validation of AL as a clinical prediction tool. We have therefore revised the Conclusion section to refer to AL as a potential risk stratification marker rather than a validated prediction tool, and we clarify that its clinical utility requires further validation in independent cohorts.

**Comment 11:** Remove or qualify the implication that reducing AL serves as a primary prevention strategy .

**Response:** We appreciate the reviewer’s important comment. We have revised the Conclusion section to remove the unqualified implication that reducing AL itself serves as a primary prevention strategy for lung cancer. Instead, we now frame AL as a marker of multisystem physiological dysregulation that may help identify individuals with elevated risk. We further clarify that whether modifying AL-related biological, behavioral, or socioeconomic factors can reduce lung cancer risk remains to be evaluated in future interventional studies.

## Responses to Reviewer #2

**Comment 1:** The authors refer to "baseline" for the follow-up, without defining it first. How is "baseline" defined? Is it date of recruitment to UKB? Date of birth? Discuss the meaning of this baseline also regarding its implication on potential biases and interpretation of the results.

**Response:** We have revised the Methods section to clearly define baseline as the date of UK Biobank enrollment, at which exposures and covariates were assessed. We also added a statement discussing the implications of this definition for result interpretation, including the possibility of baseline exposure misclassification and residual confounding inherent to observational cohort studies.

**Comment 2:** Explicitly define RERI, AP, and Synergy Index; explain how they are obtained from Cox models.

**Comment 3:** In the same regard, consider reporting the interaction hazard ratios.

**Response:** We have revised the Statistical Analysis section to explicitly define the relative excess risk due to interaction (RERI), attributable proportion due to interaction (AP), and synergy index (SI). We also clarified that these additive interaction measures were derived from hazard ratios estimated using Cox proportional hazards models across joint categories of AL and PRS.

**Comment 4:** Smoking is known to be a risk factor for lung cancer. Why was it also considered with an additional analysis?

**Response:** We thank the reviewer for raising this important point. We agree that the stronger association observed among smokers may partly reflect smoking-related systemic injury, including chronic inflammation and pulmonary damage. To address this concern, we performed additional sensitivity analyses with further adjustment for smoking-related covariates, including smoking duration, number of cigarettes smoked per day, and years since quitting smoking. After these additional adjustments, the association between AL and lung cancer was attenuated, particularly for the low AL group, which no longer reached statistical significance. However, the association remained significant for continuous AL score (HR = 1.039, 95% CI: 1.018–1.061, P < 0.001) and for the high AL group (HR = 1.370, 95% CI: 1.114–1.684, P = 0.003). These findings suggest that part of the AL–lung cancer association may overlap with smoking-related systemic damage, but the persistence of the association for high AL indicates that AL is unlikely to be merely a surrogate for smoking exposure. We have revised the Results and Discussion sections to clarify this interpretation and to acknowledge the possibility of residual confounding by smoking-related damage.

**Comment 5:** Consider adding identified proteins to the Cox models, to obtain their marginal contribution to the risk.

**Response:**We thank the reviewer for the suggestion. We agree that including these proteins in the Cox model could help evaluate their marginal associations with lung cancer risk. However, this analysis was not conducted in the present study, mainly for three reasons:

First, these proteins were selected based on their associations with AL and may lie downstream of AL-related physiological dysregulation. Including them in the main AL–lung cancer Cox model could lead to overadjustment, shifting the model’s interpretation from “the overall association between AL and lung cancer” to “the residual association after controlling for these potential intermediate proteins.”

Second, the proteomic analysis was performed in a smaller subset of UKB-PPP, with even fewer lung cancer events. Simultaneously including a large number of correlated proteins in the Cox model could easily result in overfitting, multicollinearity, and unstable estimates.

Third, the goal of this study was to explore AL-related biological features and potential protein pathways, rather than to develop or validate a protein-based prediction model. Therefore, we acknowledge in the Discussion that future studies with larger proteomic cohorts are needed to further evaluate the independent and incremental contributions of these proteins to lung cancer risk prediction.

**Comment 6:** Statistically significant physical and functional associations were identified within the network using a PPI enrichment threshold of p< 1.0 × 10⁻⁶. – Why not using more established methods for multiple testing in omics analyses, e.g. FDR controlling procedures?

**Response:** We thank the reviewer for this important comment. We agree that false discovery rate control is more appropriate for omics-scale enrichment analyses involving multiple statistical tests. In the revised manuscript, we have clarified that the STRING-reported PPI enrichment p-value was used only as a network-level measure to assess whether the observed interactions among the selected proteins were greater than expected by chance. It was not used as the sole criterion for declaring individual pathways or functional categories significant. For downstream functional enrichment analyses, including GO and KEGG analyses, we applied Benjamini–Hochberg false discovery rate correction, with FDR < 0.05 considered statistically significant. We have revised the Methods section to distinguish the global PPI enrichment test from FDR-controlled functional enrichment analyses.

**Comment 7:** How was mediation analysis conducted Please specify.

**Response:** We thank the reviewer for requesting additional details on the mediation analysis. We have revised the Methods section. The relevant modifications have been made in Lines 406-420.

“Each identified protein was assessed separately as a potential mediator. In each mediation model, AL was specified as the exposure, the protein level as the mediator, and incident lung cancer as the outcome. The mediator model estimated the association between AL and protein levels, whereas the outcome model estimated the association of AL and the protein mediator with lung cancer risk. Both models were adjusted for the same covariates used in the primary analysis. The total effect of AL on lung cancer risk was partitioned into the natural direct effect and the natural indirect effect through each protein mediator. The proportion mediated was calculated as the indirect effect divided by the total effect. Bootstrap resampling with 1,000 replicates was used to estimate 95% confidence intervals for the indirect effect and proportion mediated. Multiple testing across proteins was controlled using FDR correction, and proteins with FDR < 0.05 were considered to have evidence of mediation.”

**Result**

**Comment 8:** Remove non-significant interactions from Figure S4 and recalculate the model.

**Response:** We have revised Figure S4 by removing the non-significant interaction results, as requested. We also recalculated the corresponding regression models to ensure that the updated figure accurately reflects the revised analysis and improves the clarity of the subgroup findings.

**Comment 9:** Kaplan-Meier plots: no need to leave the event marks, the fact that they present all along the lines, causes the line to be unnecessarily thick.
**Response:** We have regenerated the Kaplan–Meier plots without event/censoring marks along the curves. This revision reduces visual clutter and improves the readability of the survival curves.

## Responses to Reviewer #3

**Comment 1:** Weak digital health relevance; benchmark against established lung cancer risk models; evaluate predictive performance (C-index, AUC, calibration); propose digital risk calculator or decision-support pathway.

**Response:** We appreciate this critical comment on digital health relevance. We have substantially revised the manuscript to strengthen its alignment with PLOS Digital Health scope. We benchmarked AL against established lung cancer screening criteria (USPSTF 2021, NLST) and traditional risk prediction models, and formally evaluated the incremental predictive value of adding AL to these models using C-index. We found that adding AL improved the discrimination of rule-based screening eligibility models.The specific modifications are as follows:

To evaluate the relevance of AL for LDCT screening risk stratification, we constructed screening eligibility variables according to the USPSTF 2021 and NLST criteria. USPSTF 2021 eligibility was defined as age 50–80 years, a smoking history of at least 20 pack-years, and current smoking or smoking cessation within the past 15 years [25]. NLST eligibility was defined as age 55–74 years, a smoking history of at least 30 pack-years, and current smoking or smoking cessation within the past 15 years [26]. Participants who did not meet the corresponding eligibility criteria were classified as screening-ineligible. To determine whether AL could refine risk stratification beyond rule-based LDCT screening eligibility, we evaluated the association between continuous AL score and lung cancer risk separately within screening-eligible and screening-ineligible groups. We further cross-classified participants according to screening eligibility status and AL category, using screening-ineligible individuals with normal AL as the reference group. Cox proportional hazards models were then used to estimate hazard ratios and 95% confidence intervals across joint exposure categories, with adjustment for the same covariates as in the primary analysis. To assess whether AL improved risk discrimination beyond established LDCT screening eligibility criteria, we compared Cox models including screening eligibility alone with models including screening eligibility plus AL. Model discrimination was evaluated using Harrell’s C-index, and changes in C-index were calculated by subtracting the C-index of the base model from that of the AL-enhanced model. In parallel, we evaluated the incremental value of AL beyond a multivariable clinical model that included demographic, clinical, and smoking-related covariates. Model improvement was assessed using likelihood ratio tests, and two-sided P values were reported.

**Reference**

[25] US Preventive Services Task Force, Krist AH, Davidson KW, Mangione CM, Barry MJ, Cabana M, et al. Screening for lung cancer: US Preventive Services Task Force recommendation statement. JAMA. 2021;325(10):962-70.

[26] Aberle DR, Adams AM, Berg CD, Black WC, Clapp JD, Fagerstrom RM, et al. Reduced lung-cancer mortality with low-dose computed tomographic screening. N Engl J Med. 2011;365(5):395-409.

**Comment 2:** Clarify incremental novelty relative to prior work (UK Biobank AL-lung cancer study, AL-PRS in colorectal cancer, AL-proteomics in kidney cancer).

**Response:** We thank the reviewer for this comment. We agree that previous studies have reported an association between AL and lung cancer risk, including analyses based on the UK Biobank. We have revised the manuscript to clarify that the novelty of our study does not lie solely in confirming the AL–lung cancer association, but in extending this evidence in several clinically and biologically relevant directions.

First, we evaluated the potential relevance of AL for lung cancer screening risk stratification by integrating AL with established LDCT screening eligibility criteria, including the USPSTF 2021 and NLST criteria. We found that AL further stratified lung cancer risk within both screening-eligible and screening-ineligible populations. High AL was significantly associated with increased lung cancer risk among NLST-ineligible individuals, suggesting that AL may provide additional risk information beyond stringent rule-based screening criteria. We also showed that adding AL improved the discrimination of rule-based screening eligibility models, with the C-index increasing from 0.723 to 0.755 for USPSTF 2021 and from 0.677 to 0.721 for NLST, whereas the gain was minimal when AL was added to a multivariable clinical model. These findings support the potential role of AL as a complementary risk-stratification marker for rule-based LDCT screening frameworks.

Second, we examined heterogeneity across lung cancer histological subtypes and found that the AL association was strongest for small cell lung cancer, followed by squamous cell carcinoma and adenocarcinoma. This subtype-specific analysis provides additional etiological granularity beyond overall lung cancer risk.

Third, we evaluated the joint effect of AL and genetic susceptibility using a lung cancer PRS. Although no significant additive interaction was detected, individuals with both high genetic risk and high AL had the highest lung cancer risk, suggesting that AL may complement genetic risk stratification.

Fourth, we incorporated plasma proteomic data to investigate biological correlates and potential mediators of AL. Using a machine learning–based feature selection and SHAP interpretation framework, we identified 111 AL-related proteins and further linked these proteins to lipid metabolism, inflammation, cytokine signaling, PI3K–Akt/MAPK pathways, and mediation of lung cancer risk. This multi-layered analysis extends the study from epidemiological association toward biological interpretation.

We have revised the Introduction and Discussion sections to more explicitly state these incremental contributions and to avoid implying that the AL–lung cancer association itself is entirely novel.

**Comment 3:** The interpretation of AL requires more caution. It is important to note that AL should not be interpreted as an established independent carcinogenic factor based on the current observational evidence.

**Response:** We agree with this comment. We have revised the entire manuscript to use cautious language and avoid overinterpreting AL as a definite causal factor in lung carcinogenesis.

**Comment 4:** Deepen proteomic and mediation analyses; conduct subtype-specific proteomic analyses ,especially SCLC; acknowledge temporal limitations and need for validation (TCGA, HPA, single-cell data).

**Response:** We appreciate this detailed comment. We have deepened the proteomic and mediation analyses as requested:

“In the subtype-specific mediation analysis, 39 significant protein–subtype mediation signals were identified, including 15 for LUAD, 19 for SCC, and 5 for SCLC. A common set of nine proteins showed significant indirect-effect signals for both LUAD and SCC, including HGF, IL6, GDF15, ADM, BCAN, NTRK3, NCAN, ACAN, and MSTN, suggesting partially shared protein-related pathways across NSCLC subtypes. For SCLC, five proteins showed significant indirect effects, including GDF15, LECT2, KITLG, BCAN, and CDH2. Among these, GDF15, LECT2, KITLG, and BCAN showed positive indirect-effect patterns, with estimated mediated proportions of 61.0%, 30.3%, 30.8%, and 10.4%, respectively. CDH2 showed an inverse indirect effect, suggesting a potentially opposing or compensatory pathway.”

**Comment 5:** Conduct detailed smoking-related sensitivity analyses (subgroup-specific, especially SCLC); incorporate smoking duration, pack-years; discuss AL may reflect smoking-related inflammatory burden.

**Response:** We thank the reviewer for raising this important point. We agree that the stronger association observed among smokers may partly reflect smoking-related systemic injury, including chronic inflammation and pulmonary damage. To address this concern, we performed additional sensitivity analyses with further adjustment for smoking-related covariates, including smoking duration, number of cigarettes smoked per day, and years since quitting smoking. After these additional adjustments, the association between AL and lung cancer was attenuated, particularly for the low AL group, which no longer reached statistical significance. However, the association remained significant for continuous AL score (HR = 1.039, 95% CI: 1.018–1.061, P < 0.001) and for the high AL group (HR = 1.370, 95% CI: 1.114–1.684, P = 0.003). These findings suggest that part of the AL–lung cancer association may overlap with smoking-related systemic damage, but the persistence of the association for high AL indicates that AL is unlikely to be merely a surrogate for smoking exposure. We have revised the Results and Discussion sections to clarify this interpretation and to acknowledge the possibility of residual confounding by smoking-related damage.

**Table 1. Baseline characteristics stratified by group**

| **Characteristics** | Excluded (n = 123,846) | Included (n = 336,932) | **SMD** |
| --- | --- | --- | --- |
| Age, mean (SD) | 57.17 (7.99) | 56.53 (8.06) | 0.080 |
| Sex, n (%) |  |  | 0.049 |
| Female | 69,362 (56.0) | 180,466 (53.6) |  |
| Male | 54,484 (44.0) | 156,466 (46.4) |  |
| BMI, mean (SD) | 28.03 (8.15) | 27.51 (5.58) | 0.074 |
| Ethnic, n (%) |  |  | 0.010 |
| Non-European | 1,629 (1.3) | 4,836 (1.4) |  |
| European | 121,762 (98.3) | 330,851 (98.2) |  |
| Smoking status, n (%) |  |  | 0.013 |
| Never | 66,334 (53.6) | 181,290 (53.8) |  |
| Former | 44,160 (35.7) | 118,604 (35.2) |  |
| Current | 12,773 (10.3) | 35,598 (10.6) |  |
| Alcohol frequency, n (%) |  |  | 0.029 |
| Daily or almost daily | 25,283 (20.4) | 71,294 (21.2) |  |
| Three or four times a week | 29,021 (23.4) | 80,328 (23.8) |  |
| Once or twice a week | 32,591 (26.3) | 87,978 (26.1) |  |
| One to three times a month | 13,849 (11.2) | 37,531 (11.1) |  |
| Special occasions only | 13,885 (11.2) | 36,192 (10.7) |  |
| Never | 9,022 (7.3) | 23,118 (6.9) |  |
| Townsend deprivation index, mean (SD) | -1.24 (4.70) | -1.34 (4.51) | 0.021 |
| Education, n (%) |  |  | 0.038 |
| College or university degree | 82,237 (66.4) | 225,574 (66.9) |  |
| Others | 39,885 (32.2) | 108,055 (32.1) |  |
| IPAQ, n (%) |  |  | 0.019 |
| Low | 18,741 (15.1) | 51,035 (15.1) |  |
| Moderate | 40,884 (33.0) | 111,372 (33.1) |  |
| High | 40,046 (32.3) | 111,072 (33.0) |  |
| Family history of cancer, n (%) |  |  | 0.029 |
| No | 70,756 (57.1) | 197,153 (58.5) |  |
| Yes | 45,403 (36.7) | 119,011 (35.3) |  |
| PRS group, n (%) |  |  | <0.001 |
| Low (≤25%) | 30,962 (25.0) | 84,233 (25.0) |  |
| Intermediate (26–75%) | 61,922 (50.0) | 168,466 (50.0) |  |
| High (>75%) | 30,962 (25.0) | 84,233 (25.0) |  |
| Note: Data are presented as mean (SD) or n (%). SMD, standardized mean difference; BMI, body mass index; IPAQ, International Physical Activity Questionnaire; CCVD, cardiovascular and cerebrovascular disease; CVD, cardiovascular disease; PRS, polygenic risk score. | | | |
