# Supplementary material for: Allostatic load, genetic susceptibility, and lung cancer risk: Evidence from a prospective cohort and integrative proteomic analyses
Source: PLOS Digit Health. 2026 Oct 14;5(10):e0001766. doi: 10.1371/journal.pdig.0001766 (PMC13667778; doi:10.1371/journal.pdig.0001766)
Supplement: Supplementary file 21 [file pdig.0001766.s026.docx]

**Response to Reviewers**

Dear Editor and Reviewers,

We sincerely thank the Editor and Reviewers for their careful evaluation of our revised manuscript entitled “Allostatic Load, Genetic Susceptibility, and Lung Cancer Risk: Evidence from a Prospective Cohort and Integrative Proteomic Analyses.” We greatly appreciate the positive and constructive comments provided during this round of review.

We are particularly grateful that both reviewers considered our previous concerns to have been adequately addressed and that the methodological rigor, statistical analyses, data availability, and overall presentation of the manuscript were viewed favorably. We also appreciate Reviewer #3’s recognition of the revisions made to strengthen the digital health relevance of the study, clarify the incremental novelty of our work, refine the interpretation of the proteomic and mediation analyses, and provide additional smoking-related sensitivity and subtype-specific analyses.

In response to the remaining minor concerns, we carefully reviewed the entire manuscript and performed further editorial refinement. We revised several expressions that could potentially imply causal or mechanistic interpretations beyond what can be supported by the observational study design. We also further clarified the interpretation of the proteomic and mediation analyses. These findings are now consistently presented as exploratory and hypothesis-generating rather than as evidence of established causal biological mediation. Correspondingly, the Discussion has been revised to emphasize that the identified proteins and enriched pathways provide biological context for the observed association between allostatic load and lung cancer risk, while causal relationships cannot be established from the present observational analyses.

In addition, we refined the description of allostatic load throughout the manuscript to consistently characterize it as a composite marker of multisystem physiological dysregulation and physiological vulnerability. We also revised the wording related to genetic susceptibility and low-dose computed tomography screening analyses to more accurately describe the incremental risk information provided by allostatic load beyond established screening eligibility criteria, without overstating its current clinical applicability.

Finally, we carefully proofread the manuscript and corrected grammatical, stylistic, and terminological inconsistencies to improve clarity and readability. These revisions do not alter the main analyses or conclusions of the study but further align the interpretation of the findings with the observational nature of the study and the reviewers’ recommendations.

We sincerely appreciate the thoughtful comments and constructive guidance from the Editor and Reviewers. We believe that these final revisions have further improved the clarity, rigor, and balance of the manuscript, and we hope that the revised version is now suitable for publication in PLOS Digital Health.

Sincerely yours,

Xiaoxiao Wang, PhD

Clinical Research Institute

Affiliated Hospital of Nanjing University of Chinese Medicine (Jiangsu Hospital of Traditional Chinese Medicine)

155 Hanzhong Road

Qinhuai District

Nanjing

China

Phone: +86 13770784000

Email: yfy106@njucm.edu.cn
