# Supplementary material for: Allostatic load, genetic susceptibility, and lung cancer risk: Evidence from a prospective cohort and integrative proteomic analyses
Source: PLOS Digit Health. 2026 Oct 14;5(10):e0001766. doi: 10.1371/journal.pdig.0001766 (PMC13667778; doi:10.1371/journal.pdig.0001766)
Supplement: Peer review history

# Peer review: Decision Letter 0

**DOI:** 10.1371/journal.pdig.0001766.r001

**Authors:** Laura Sbaffi, Junhan Zhao

28 Apr 2026

Response to Reviewers Revised Manuscript with Track Changes Manuscript **Journal Requirements:**

1. Please update your online Competing Interests statement. If you have no competing interests to declare, please state: "The authors have declared that no competing interests exist."

2. In the online submission form, you indicated that:

"The study is based on the UK Biobank data. The UK Biobank is an open-access resource, and eligible researchers can obtain the data by registering and applying at http://ukbiobank.ac.uk/register-apply/. Other data or codes that support the findings of this study are available from the corresponding author upon reasonable request."

All PLOS journals now require all data underlying the findings described in their manuscript to be freely available to other researchers, either

a) In a public repository,

b) Within the manuscript itself, or

d) Uploaded as supplementary information.

This policy applies to all data except where public deposition would breach compliance with the protocol approved by your research ethics board. If your data cannot be made publicly available for ethical or legal reasons (e.g., public availability would compromise patient privacy), please explain your reasons by return email and your exemption request will be escalated to the editor for approval. Your exemption request will be handled independently and will not hold up the peer review process, but will need to be resolved should your manuscript be accepted for publication. One of the Editorial team will then be in touch if there are any issues.

For further assistance, you may go to: http://journals.plos.org/digitalhealth/s/data-availability

3. If the reviewer comments include a recommendation to cite specific previously published works, please review and evaluate these publications to determine whether they are relevant and should be cited. There is no requirement to cite these works unless the editor has indicated otherwise.

**Additional Editor Comments (if provided):**

**Reviewers' Comments:**

**Comments to the Author**

1. Does this manuscript meet PLOS Digital Health’s publication criteria?

Reviewer #1: Yes

Reviewer #2: Yes

Reviewer #3: Partly

**********

2. Has the statistical analysis been performed appropriately and rigorously?

Reviewer #1: Yes

Reviewer #2: Yes

Reviewer #3: Yes

**********

3. Have the authors made all data underlying the findings in their manuscript fully available (please refer to the Data Availability Statement at the start of the manuscript PDF file)?

The PLOS Data policy

Reviewer #1: Yes

Reviewer #2: Yes

Reviewer #3: No

**********

4. Is the manuscript presented in an intelligible fashion and written in standard English?

Reviewer #1: Yes

Reviewer #2: Yes

Reviewer #3: Yes

**********

Reviewer #1: REVIEW

Abstract

This abstract addresses an important question and is strengthened by the authors' use of a large prospective cohort, subtype-specific analyses, and efforts to connect epidemiologic associations with proteomic mechanisms. That said, I suggest the causal language, specifically regarding 'mechanistic pathways' and 'linking' AL to carcinogenesis, is overreaching, as it extends beyond what observational mediation analyses can prove. I recommend tempering this phrasing. Furthermore, I suggest the authors clarify in the abstract whether smoking and related confounders were controlled for rigorously enough to truly support an independent association for AL in lung cancer risk

Introduction

The authors have drafted a well-organized introduction that logically progresses from epidemiologic burden to proteomics, clearly filling the knowledge gaps around subtype heterogeneity and joint effects. To improve the scientific rigor of this section, I recommend the following adjustments:

• I suggest toning down the language stating that plasma proteomics 'elucidate' causal pathways. I recommend the authors explicitly frame these analyses as exploratory and hypothesis-generating rather than mechanistic proof.

• I suggest a tighter treatment of AL. Rather than presenting it purely as a modifiable determinant, I recommend acknowledging it as a composite biomarker that overlaps with behavioral risk profiles.

• Given the nature of AL, I highly recommend the authors directly recognize the possibility that it functions as a correlate of cumulative disadvantage (e.g., smoking, socioeconomic factors) rather than a distinct causal driver. Acknowledging this will better align the premise with the realities of observational data

These are my concerns. Thank you.

Methods

The Methods section is well-developed and highlights several strengths, particularly the prospective cohort design, the robust sample size, and the inclusion of multiple sensitivity analyses. The effort to address confounding through adjustments for smoking, socioeconomic factors, and family history, alongside the exclusion of early events and COPD—is a thoughtful approach to common epidemiologic concerns. However, there are a few technical areas where I believe the manuscript would benefit from further clarity or justification.

Regarding the participant flow, I suggest that the authors address the exclusion of 126,829 individuals due to missing allostatic load (AL) biomarkers. This is a substantial proportion of the original cohort and naturally raises concerns about selection bias. I recommend providing a comparison table or a brief analysis to show whether those excluded differ systematically from the retained sample, which would help the reader judge the representativeness of the findings.

The construction of the AL score also warrants a more detailed rationale. While dichotomizing biomarkers by sample quartiles is a frequent practice, it remains a relatively coarse approach that can make the score highly population-dependent. Furthermore, the decision to define AL = 0 as "normal" and then split the remaining distribution by the median is somewhat unconventional. I suggest that the authors justify this specific categorization and, if possible, demonstrate whether the primary results remain robust under alternative or more clinically anchored AL definitions.

In the omics and machine learning sections, the workflow is quite ambitious, but there is a risk of overstating the findings. While the use of XGBoost, Boruta-SHAP, and mediation analysis is technically interesting, these methods are better suited for hypothesis generation than for establishing definitive biological mechanisms. I recommend being more explicit regarding the following:

• Temporality and Technical Effects: How were batch effects and multiple testing controlled for in the proteomic subset?

• Mediation Assumptions: Given the high likelihood of residual confounding from smoking intensity and preclinical disease, the assumptions required for formal mediation are difficult to satisfy in this observational context.

• Machine Learning Rigor: It would be helpful to clarify if feature selection and SHAP ranking were fully nested within cross-validation to prevent data leakage, and whether the identified proteins were validated in an independent subset.

These are my concerns, and I believe they can improve the manuscript.

Results

The Results section is well-organized, showing a consistent association between higher allostatic load (AL) and lung cancer incidence, particularly for SCLC. While the subtype-specific analyses and sensitivity tests are strengths, the interpretation requires more restraint to avoid overstating the findings.

I suggest that the authors maintain a cautious tone regarding the clinical relevance of the hazard ratios, as many are modest in magnitude. The threshold effect (AL > 3) and claims of nonlinearity may be sample-specific or artifacts of the AL score construction. Furthermore, I recommend acknowledging that stronger associations in men and current smokers may reflect residual confounding or differences in baseline risk rather than definitive biological effect modification.

The omics results are particularly susceptible to overinterpretation. Although the identified proteins provide a high-resolution profile, this remains exploratory observational work. I suggest that the authors explicitly state that proteins such as HGF, RARRES2, and GDF15 are likely markers of inflammation, smoking burden, or preclinical disease rather than proven mechanistic transmitters of chronic stress.

The large mediation proportions depend on strong observational assumptions that are unlikely to be fully satisfied. I recommend interpreting these as hypothesis-generating.

Given the multiple layers of analysis, from cohort models to proteomic feature selection, I suggest that the authors clarify how they controlled for multiplicity to mitigate the risk of false positives.

Discussion and Conclusion

The Discussion effectively contextualizes the epidemiologic findings within the frameworks of chronic inflammation and lung cancer heterogeneity. The emphasis on SCLC is particularly insightful, given its established links to inflammatory pathways. While the prospective design and large sample size provide a strong foundation, the interpretation frequently overreaches the data's limits.

I suggest that the authors avoid treating allostatic load (AL) as a biologically active driver of carcinogenesis. In this observational context, where smoking, comorbidity, and deprivation are heavily intertwined, the proteomic findings should be framed as candidate correlates rather than confirmed mediators. Specifically, the claims regarding HGF, RARRES2, and GDF15 are overstated; I recommend characterizing these as biologically suggestive signals rather than mechanistic proof.

The Conclusion accurately notes the association between AL and lung cancer incidence, but certain implications require more caution:

• Predictive Value: The statement that AL serves as a "predictive factor" pushes beyond the current evidence. Unless incremental predictive value and formal performance metrics (e.g., C-index improvement) are demonstrated over existing risk factors, I suggest that the authors refer to AL as a risk marker rather than a validated prediction tool.

• Primary Prevention: I recommend removing or heavily qualifying the implication that reducing AL serves as a primary prevention strategy. This study did not test interventions; therefore, it cannot establish that modifying AL changes risk.

I suggest that the authors refocus the conclusion on AL as a potential stratification marker that warrants further mechanistic and interventional research, rather than implying clinical readiness.

I strongly believe addressing these concerns will improve the paper.

Thank you.

Reviewer #2: Methods:

1. The authors refer to "baseline" for the follow-up, without defining it first. For instance, in "Outcome assessment": "Participants were followed from baseline until the diagnosis of lung cancer or censoring" –

how is "baseline" defined? Is it date of recruitment to UKB? Date of birth? Discuss the meaning of this baseline also regarding its implication on potential biases and interpretation of the results.

2. "the relative excess risk due to the interaction (RERI), the attributable proportion due to the interaction (AP) and Synergy Index (SI)." – please define these types of effects more explicitly, and how they can be obtained by the Cox model.

3. In the same regard, consider reporting the interaction hazard ratios.

4. "we additionally adjusted for smoking-related variables,…" - Smoking is known to be a risk factor for lung cancer. Why was it also considered with an additional analysis?

5. Consider adding identified proteins to the Cox models, to obtain their marginal contribution to the risk.

6. Statistically significant physical and functional associations were identified within the network using a PPI enrichment threshold of p< 1.0 × 10⁻⁶. – Why not using more established methods for multiple testing in omics analyses, e.g. FDR controlling procedures?

7. " Mediation analyses were conducted to evaluate the potential contribution of identified proteins to the associations between AL and lung cancer risk, with models adjusted for the same covariates. " - How was mediation analysis conducted Please specify.

Result

1. Figure S4: I don't see the use of leaving so many non-significant interactions in the model, typically those are removed, and then the model is recalculated.

2. All Kaplan-Meier plots: no need to leave the event marks, the fact that they present all along the lines, causes the line to be unnecessarily thick.

Minor comments

1. The use of the word "significantly" for statistical results is not needed, its either associated (if significant) or not.

2. "indexes" or "indices"?

Reviewer #3: Decision: Major Revision

This study is based on a large prospective cohort from the UK Biobank and evaluates the association between allostatic load (AL) and the risk of overall lung cancer as well as different histological subtypes. The authors further integrate polygenic risk score (PRS) and plasma proteomic data to explore genetic susceptibility and potential protein-mediated mechanisms. The study has a large sample size, long follow-up duration, and potential public health relevance. Specifically, the authors included 336,945 UK Biobank participants for lung cancer risk analysis and used the UKB-PPP subcohort to integrate proteomic data for mechanistic exploration. Overall, the study framework is relatively comprehensive.

However, the current manuscript appears to be more closely aligned with molecular epidemiology or cancer prevention epidemiology than with the core scope of PLOS Digital Health. Although the authors used XGBoost, SHAP, PRS, and multidimensional proteomic data, these methods are mainly used as analytical tools. The manuscript does not truly establish, validate, or present a digital tool for risk prediction, screening stratification, or clinical decision support. At present, the findings mainly identify several risk-associated factors rather than demonstrating a digital health application.

Major Comments

1. Weak digital health relevance

The authors state in the abstract that incorporating AL may improve the identification of high-risk populations for lung cancer. However, the main text primarily reports exposure–outcome association analyses, joint PRS–AL stratification, and proteomic mechanistic exploration. It does not adequately answer the key digital health question of “how high-risk individuals can be better identified.”

At present, the manuscript is more an application of data science methods than a study with clear clinical or public health translation in the digital health sense. Readers of PLOS Digital Health may be more interested in whether the proposed model can be used for screening, whether it outperforms existing lung cancer risk prediction models, whether it can assist LDCT screening eligibility decisions, and whether it is interpretable, reproducible, deployable, and externally validated.

The clinical applicability of this study would be substantially strengthened if the authors could compare their approach with existing lung cancer screening criteria or established risk prediction models; propose a digital risk calculator, algorithmic framework, or clinical decision-support pathway; or formally evaluate whether adding AL to traditional risk models improves C-index, time-dependent AUC, calibration, or other predictive performance metrics.

2. The novelty should be more clearly defined

Several studies based on large population databases such as UK Biobank and NHANES have already evaluated the role of allostatic load in cancer risk, cancer mortality, and other chronic disease outcomes. In particular, a previous UK Biobank study has directly examined the association between AL and lung cancer risk and suggested that this association was mainly observed among former and current smokers (PMID: 39335207). Therefore, the overall association between AL and lung cancer risk is not an entirely novel finding.

Similarly, the joint evaluation of AL and genetic susceptibility has been reported in colorectal cancer (PMID: 39271163), and the combination of AL with proteomic mediation analysis has also been applied in kidney cancer (PMID: 41088055). Based on this context, the novelty of the present study mainly lies in integrating AL, lung cancer histological subtypes, PRS, and proteomic mechanistic exploration within a single analytical framework. Therefore, the current contribution is primarily an incremental extension in the specific disease context of lung cancer and its histological subtypes. The authors should more clearly articulate the incremental contribution of this study relative to existing literature.

3. The interpretation of AL requires more caution

This study uses a prospective cohort design, excludes participants with cancer at baseline, and applies Cox regression models to evaluate the association between AL and lung cancer risk. However, it is important to note that AL should not be interpreted as an established independent carcinogenic factor based on the current observational evidence.

AL is a composite score constructed from biomarkers including HbA1c, HDL, LDL, total cholesterol, triglycerides. It is not a direct measure of “chronic psychological stress” or “long-term physiological stress.” Rather, it may reflect a composite state involving obesity, aging, smoking-related damage, metabolic abnormalities, inflammation, socioeconomic status, and subclinical disease. It may also represent an accompanying state of preclinical or subclinical illness.

Therefore, the authors should use more cautious language and avoid overinterpreting AL as a definite causal factor in lung carcinogenesis. It would be more appropriate to describe AL as a composite biomarker of multisystem physiological dysregulation associated with lung cancer risk.

4. The proteomic and mediation analyses lack depth, and the mechanistic conclusions are too strong

The proteomic and mediation analyses are potentially important parts of the manuscript, but their current depth is insufficient. In particular, HGF, RARRES2, and GDF15 are relatively broad cancer-related, inflammation-related, metabolic, or stress-associated proteins. They are not strongly tissue-specific markers for lung cancer. The manuscript also lacks validation at the tissue or single-cell level.

One of the major strengths of the current study is the analysis of lung cancer subtypes. However, the authors did not conduct subtype-specific proteomic analyses. This weakens the link between the proteomic findings and the most interesting disease-specific observation, namely the potentially stronger association between AL and SCLC.

Moreover, proteomic alterations may represent accompanying molecular changes related to disease status, or downstream molecular changes associated with AL, rather than true causal mediators. Because AL and proteomic measurements were assessed at baseline, the temporal order between AL, proteins, and lung cancer development is not fully established.

Therefore, the authors should avoid overly strong causal or mechanistic language. The Discussion should explicitly acknowledge that the proteomic findings are exploratory and hypothesis-generating, and that statistical mediation does not establish biological causality. Additional validation using lung cancer tissue data, TCGA datasets, Human Protein Atlas, or single-cell RNA-seq datasets would improve the mechanistic credibility of the study.

5. Smoking is a major potential confounder

Lung cancer, especially SCLC, is strongly associated with smoking. In this study, the association between AL and lung cancer risk appears strongest among current smokers, whereas no significant association is observed among never smokers. This finding is critical for interpreting the entire study.

It suggests that AL may not simply represent “chronic physiological stress,” but may also capture smoking-related systemic injury, chronic inflammation, COPD, metabolic abnormalities, and overall health deterioration. Therefore, the authors should not interpret AL as an independent stress-related carcinogenic factor without sufficient caution.

The authors should perform more detailed sensitivity analyses in smoking subgroups. In particular, smoking-adjusted sensitivity analyses should be performed specifically for SCLC. If available, smoking duration, cigarettes per day, years since quitting, or pack-years should be incorporated more explicitly. The authors should also discuss that AL may partly reflect smoking-related inflammatory burden rather than a completely smoking-independent chronic stress effect.

Overall recommendation for improvement

Overall, If the authors can supplement analyses demonstrating the incremental value of AL in traditional lung cancer risk prediction models, further address smoking-related confounding, strengthen the SCLC-specific findings, deepen and validate the proteomic analyses, and substantially improve the manuscript’s fit with the digital health scope, the study may still have value for further review and revision.

Minor Comments

1.The quality of the figures should be substantially improved. Several figures have low resolution, small fonts, cropping issues, or limited readability.

2.The data and code availability statement is insufficient.

3.Several expressions and grammatical issues require correction. The manuscript would benefit from systematic language editing, especially in the abstract, figure legends, table footnotes, and methods section.

**********

what does this mean?). If published, this will include your full peer review and any attached files.

**Do you want your identity to be public for this peer review?**  If you choose “no”, your identity will remain anonymous but your review may still be made public.

For information about this choice, including consent withdrawal, please see our Privacy Policy

Reviewer #1: **Yes:**  Victor Ifechukwude Agboli

Reviewer #2: **Yes:**  Anat Reiner-Benaim

Reviewer #3: No

**********

**Figure resubmission:**

**Reproducibility:**

To enhance the reproducibility of your results, we recommend that authors of applicable studies deposit laboratory protocols in protocols.io, where a protocol can be assigned its own identifier (DOI) such that it can be cited independently in the future. Additionally, PLOS ONE offers an option to publish peer-reviewed clinical study protocols. Read more information on sharing protocols at https://plos.org/protocols?utm_medium=editorial-email&utm_source=authorletters&utm_campaign=protocols

# Author comment: Author response to Decision Letter 1

**DOI:** 10.1371/journal.pdig.0001766.r002

12 Jun 2026

Attachment Submitted filename: Response to Reviewers.docx

# Peer review: Decision Letter 1

**DOI:** 10.1371/journal.pdig.0001766.r003

**Authors:** Laura Sbaffi, Junhan Zhao

5 Aug 2026

Response to ReviewersRevised Manuscript with Track ChangesManuscript**Journal Requirements:** **Additional Editor Comments (if provided):** **Reviewers' Comments:**

**Comments to the Author**

Reviewer #2: All comments have been addressed

Reviewer #3: All comments have been addressed

**********

publication criteria?

Reviewer #2: Yes

Reviewer #3: Yes

**********

3. Has the statistical analysis been performed appropriately and rigorously?

Reviewer #2: Yes

Reviewer #3: Yes

**********

4. Have the authors made all data underlying the findings in their manuscript fully available (please refer to the Data Availability Statement at the start of the manuscript PDF file)?

The PLOS Data policy

Reviewer #2: Yes

Reviewer #3: Yes

**********

5. Is the manuscript presented in an intelligible fashion and written in standard English?

Reviewer #2: Yes

Reviewer #3: Yes

**********

Reviewer #2: All comments were properly addressed.

Reviewer #3: I thank the authors for their detailed and constructive responses to my previous comments. Overall, I appreciate the substantial efforts made in revising the manuscript. The authors have addressed my major concerns in a thoughtful manner.

1. The authors have strengthened the digital health relevance of the study by comparing AL with established LDCT screening eligibility criteria, including USPSTF 2021 and NLST, and by evaluating whether AL improves risk stratification beyond these frameworks.

2. The authors have clarified the incremental novelty of the study relative to previous work on AL and cancer risk. The contribution now lies not merely in confirming the association between AL and lung cancer, but in integrating lung cancer subtype analyses, PRS-based risk stratification, proteomic exploration, and screening-related risk 3. The authors have appropriately toned down causal and mechanistic language throughout the manuscript. Reframing AL as a composite biomarker of multisystem physiological dysregulation, rather than an established causal factor in lung carcinogenesis, is more consistent with the observational nature of the study. Similarly, the proteomic and mediation findings are now more appropriately presented as exploratory and hypothesis-generating.

4. The additional smoking-related sensitivity analyses are helpful and improve the credibility of the findings. Although residual confounding related to smoking-associated injury cannot be fully excluded, the authors have now acknowledged this limitation more clearly and interpreted the results with greater caution.

5. The newly added subtype-specific mediation analyses further strengthen the manuscript by linking proteomic findings more directly with lung cancer histological heterogeneity. Although these analyses should still be interpreted cautiously, they provide useful biological context and improve the internal coherence of the study.

Overall, I believe the authors have made meaningful revisions that improve the rigor, clarity, and journal fit of the manuscript. The remaining issues are minor and can be addressed through careful wording and final editorial refinement.

**********

what does this mean?). If published, this will include your full peer review and any attached files.

**Do you want your identity to be public for this peer review?**  If you choose “no”, your identity will remain anonymous but your review may still be made public.

For information about this choice, including consent withdrawal, please see our Privacy Policy

Reviewer #2: No

Reviewer #3: No

**********

**Figure resubmission:**

**Reproducibility:**

To enhance the reproducibility of your results, we recommend that authors of applicable studies deposit laboratory protocols in protocols.io, where a protocol can be assigned its own identifier (DOI) such that it can be cited independently in the future. Additionally, PLOS ONE offers an option to publish peer-reviewed clinical study protocols. Read more information on sharing protocols at https://plos.org/protocols?utm_medium=editorial-email&utm_source=authorletters&utm_campaign=protocols

# Author comment: Author response to Decision Letter 2

**DOI:** 10.1371/journal.pdig.0001766.r004

22 Aug 2026

Attachment Submitted filename: Response_to_Reviewers_auresp_2.docx

# Peer review: Decision Letter 2

**DOI:** 10.1371/journal.pdig.0001766.r005

**Authors:** Laura Sbaffi, Junhan Zhao

20 Sep 2026

Allostatic Load, Genetic Susceptibility, and Lung Cancer Risk: Evidence from a Prospective Cohort and Integrative Proteomic Analyses

PDIG-D-26-00320R2

Dear Dr. Wang,

We are pleased to inform you that your manuscript 'Allostatic Load, Genetic Susceptibility, and Lung Cancer Risk: Evidence from a Prospective Cohort and Integrative Proteomic Analyses' has been provisionally accepted for publication in PLOS Digital Health.

Before your manuscript can be formally accepted you will need to complete some formatting changes, which you will receive in a follow-up email from a member of our team.

Please note that your manuscript will not be scheduled for publication until you have made the required changes, so a swift response is appreciated.

IMPORTANT: The editorial review process is now complete. PLOS will only permit corrections to spelling, formatting or significant scientific errors from this point onwards. Requests for major changes, or any which affect the scientific understanding of your work, will cause delays to the publication date of your manuscript.

If your institution or institutions have a press office, please notify them about your upcoming paper to help maximize its impact. If they'll be preparing press materials, please inform our press team as soon as possible -- no later than 48 hours after receiving the formal acceptance. Your manuscript will remain under strict press embargo until 2 pm Eastern Time on the date of publication. For more information, please contact digitalhealth@plos.org.

Thank you again for supporting Open Access publishing; we are looking forward to publishing your work in PLOS Digital Health.

Best regards,

Junhan Zhao

Academic Editor

PLOS Digital Health

***********************************************************

**Additional Editor Comments (if provided):**

**Reviewer Comments (if any, and for reference):**

Reviewer's Responses to Questions

**Comments to the Author**

Reviewer #2: All comments have been addressed

Reviewer #3: All comments have been addressed

**********

publication criteria?

Reviewer #2: Yes

Reviewer #3: Yes

**********

3. Has the statistical analysis been performed appropriately and rigorously?

Reviewer #2: (No Response)

Reviewer #3: Yes

**********

4. Have the authors made all data underlying the findings in their manuscript fully available (please refer to the Data Availability Statement at the start of the manuscript PDF file)?

The PLOS Data policy

Reviewer #2: Yes

Reviewer #3: Yes

**********

5. Is the manuscript presented in an intelligible fashion and written in standard English?

Reviewer #2: Yes

Reviewer #3: Yes

**********

**Reviewer #2:**  All comcerns have been addressed.

**Reviewer #3:**  My main scientific concerns have been adequately addressed, and I recommend acceptance for publication. Before finalizing the manuscript, please ensure consistency in the reporting of follow-up times and verify the accuracy and consistency of the references cited for the PRS methodology.

**********

what does this mean?). If published, this will include your full peer review and any attached files.

**Do you want your identity to be public for this peer review?**  If you choose “no”, your identity will remain anonymous but your review may still be made public.

For information about this choice, including consent withdrawal, please see our Privacy Policy

Reviewer #2: No

Reviewer #3: No

**********
